# HIV-1 adaptation to NK cell-mediated immune pressure

**DOI:** 10.1371/journal.ppat.1006361

**Published:** 2017-06-05

**Authors:** Marjet Elemans, Lies Boelen, Michael Rasmussen, Søren Buus, Becca Asquith

**Affiliations:** 1 Section of Immunology, Imperial College London, London, United Kingdom; 2 Department of Immunology and Microbiology, University of Copenhagen, Copenhagen, Denmark; Emory University, UNITED STATES

## Abstract

The observation, by Alter *et al*., of the enrichment of NK cell “escape” variants in individuals carrying certain Killer-cell Immunoglobulin-like Receptor (KIR) genes is compelling evidence that natural killer (NK) cells exert selection pressure on HIV-1. Alter *et al* hypothesise that variant peptide, in complex with HLA class I molecules binds KIR receptors and either increases NK cell inhibition or decreases NK cell activation compared to wild type peptide thus leading to virus escape from the NK cell response. According to this hypothesis, in order for NK cells to select for an escape variant, an individual must carry both the KIR and an HLA ligand that binds the variant peptide. In this study we estimate the proportion of the population that is capable of selecting for escape variants and use both epidemiological modelling and a model-free approach to investigate whether this proportion explains the observed variant enrichment. We found that the fraction of individuals within whom the variant would have a selective advantage was low and was unable to explain the high degree of enrichment observed. We conclude that whilst Alter *et al*’s data is consistent with selection pressure, the mechanism that they postulate is unlikely. The importance of this work is two-fold. Firstly, it forces a re-evaluation of some of the clearest evidence that NK cells exert a protective effect in HIV-1 infection. Secondly, it implies that there is a significant aspect of immunology that is not understood: it is possible that KIRs bind much more widely than was previously appreciated; that a gene in linkage with the KIR genes is responsible for considerable peptide-dependent selection or that variant peptides are indirectly impacting KIR ligation.

## Author summary

In our opinion, some of the best evidence that natural killer (NK) cells contribute to the control of HIV-1 infection is the identification of 22 virus variants, “KIR footprints”, that were hypothesised to escape the NK cell response (Nature, 2011). The authors (Alter, Heckerman, Schneidewind, Fadda *et al*) postulate that the variant peptide in complex with HLA class I molecules modulates the KIR signal and thus reduces recognition of the virus-infected cell by NK cells. Under this hypothesis, a viral variant can only escape the KIR-mediated NK cell response if both the NK receptor (KIR) and its ligand—an HLA molecule—are present within the host and the viral variant binds to the HLA molecule. Here we show that this triple requirement severely limits the number of hosts in which a given virus variant has a selective advantage and, as a result, the authors’ hypothesis is inconsistent with their reported data. These findings imply that there is a significant aspect of NK cell immunobiology that we do not understand and calls for a re-evaluation of the assumption that KIR footprints are evidence for a protective effect of NK cells.

## Introduction

Natural killer (NK) cells mediate anti-viral immunity by lysing infected cells, producing pro-inflammatory cytokines and modulating adaptive immunity. NK cells express activating and inhibitory receptors; the balance of signals from these receptors determines the NK cell response. Some of the best studied NK cell receptors are the Killer-cell Immunoglobulin-like Receptors (KIRs). KIRs are a polymorphic, polygenic family that includes both inhibitory and activating forms [1]. The ligands for the KIRs include the classical histocompatibility leucocyte antigen (HLA) class I molecules, which are recognised in broad allotypes. KIRs exhibit a degree of peptide specificity as amino acid variation in the C-terminal end of peptides presented by the HLA ligands has been shown to modulate KIR signalling [2–8].

Mounting epidemiological and functional evidence suggests that NK cells play an important role in the control of human immunodeficiency virus-1 (HIV-1) infection. Firstly, HIV-1 Nef protein has been shown to downregulate ligands for activating NK receptors from the surface of infected cells (including MICA, ULBP1 and ULBP2 [9–11]). Secondly, soluble ligands for activating receptors which impair NK cell-mediated cytotoxicity are released during HIV-1 infection [12]. Thirdly, gene association studies show that the gene encoding the NK receptor KIR3DL1 with the gene for its ligand Bw4-80I and of the gene encoding KIR3DS1 with its putative ligand Bw4-80I are associated with slower rates of disease progression [13–15] and reduced risk of infection [16, 17]. Finally, antibody dependent cellular cytotoxicity [18] was associated with a modest protective effect (odds ratio<1, but not statistically significant) in the RV144 vaccine trial [18].

More recently, Alter *et al* studied the relationship between KIR genotype and HIV-1 sequence in a cohort of HIV-1-infected individuals [19]. They identified 22 positions in the HIV-1 genome at which amino acid polymorphisms were significantly associated with the presence of specific KIR genes, so-called “KIR-footprints”. They focussed on 6 viral sequence polymorphisms enriched in *KIR2DL2*^*+*^ individuals compared to *KIR2DL2*^*–*^ individuals. *In vitro*, variant virus replicates more rapidly than wild type virus in the presence of NK cells from *KIR2DL2*^*+*^ donors. Alter and co-authors [19–22] postulate that the variant peptide modulates the KIR signal (increasing inhibition for inhibitory KIRs and decreasing activation for activating KIRs) thus reducing recognition of variant virus-infected cells by NK cells expressing the relevant KIR. That is, the variant virus confers escape from NK cells and is selected for, analogous to the well-documented phenomenon of HIV-1 escape from CD8^+^ T cells [23–25]. In summary, Alter *et al* demonstrate that NK cells exert selection pressure on HIV-1 and thus provide strong evidence that NK cells have direct antiviral effector function *in vivo*.

However, in order to modulate NK cell activity, the hypothesis of Alter *et al* requires that both the KIR receptor and its HLA class I ligand are present. The genes for HLA and KIR are on different chromosomes and segregate independently [1]. Consequently, a fraction of the population will have a KIR but will be missing the HLA ligand. Furthermore, for a virus polymorphism to have an inhibitory effect on NK cell function, the variant peptide needs to be presented by the HLA ligand. A priori, it is not obvious that a sufficient number of people will meet the triple requirement of having the relevant KIR and having an HLA molecule that both ligates the KIR and binds the variant peptide. The aim of this study was to quantify the fraction of the population that meets this triple requirement and then to ascertain whether this fraction is sufficient to explain the degree of selection observed in the population. The goal is to investigate the hypothesis put forward by Alter and co-authors to explain KIR footprints in HIV-1 sequence and whether such selection would be detected in the population, it is not to investigate the role of NK cells in the control of HIV-1 in general.

Using both *in silico* epitope prediction (with two independent algorithms) and an *in vitro* peptide:HLA stability assay we found that the fraction of the population that was capable of exerting selection pressure for variant virus was low. Epidemiological modelling shows that this low fraction of selectors was unable to explain the high degree of enrichment observed. A simple, model-free approach confirmed this result. We conclude that Alter *et al*’s data is consistent with selection pressure but that the mechanism that they postulate is unlikely. HLA class I molecules bind diverse peptides; not enough different HLA molecules bind the same viral peptide to drive the observed variant enrichment. This calls for a re-evaluation of what was apparently some of the clearest evidence that NK cells exert a selective effect in HIV-1 infection. Furthermore, it suggests that there is a significant aspect of immunology that we do not understand: either KIRs bind HLA:peptide much more widely than is currently appreciated; or a gene in linkage with the KIR genes is responsible for significant peptide-dependent selection; or variant peptides are indirectly impacting KIR ligation.

## Results

### Variant virus has a selective advantage in a small fraction of the population

We initially focussed on the 4 viral variants (6 amino acid polymorphisms) studied in depth in [19]. All 4 variants are enriched in *KIR2DL2*^*+*^ individuals. One of the variant strains, Vpu(71M/74H) overlaps with Env(17W/20M); there are thus three independent variants: Gag(138L), Nef(9S) and Vpu(71M/74H)-Env(17W/20M). KIR2DL2 is an inhibitory receptor. It is postulated that the variant peptide causes stronger inhibitory signalling via KIR2DL2 than wild type [19–22]. This model requires that the variant peptide binds one or more of the KIR2DL2-ligating HLA class I molecules and that the amino acid polymorphism either enhances KIR signalling (i.e. the polymorphism affects KIR-peptide contact) and/or enhances peptide-HLA binding (i.e. the polymorphism affects ligand availability). We define individuals who carry both HLA class I and KIR alleles that meet these conditions as ‘selectors’, individuals where, according to our current understanding of NK cell activation, the variant virus could have a selective advantage. KIR2DL2 binds HLA C molecules which have an asparagine at position 80 (designated the C1 group of alleles), HLA-B*46:01 and B*73:01 (which have a HLA C-type motif at residues 77–83), and with weaker affinity, HLA C molecules with a lysine at position 80 (C2 group alleles) [26]. KIRs contact the C-terminal end (specifically positions PC-1 and PC-2) of the bound peptide [3, 5, 6, 27–29]. We calculated the proportion of selectors in the HIV-1-infected population in the USA (the population studied by Alter *et al* [19]), by using the HLA-gene frequency in different ethnic groups [30], the frequency of these groups in the HIV-1-infected population [31] and the binding affinity of 8- to 11mer variant peptides estimated using NetMHCpan v2.8 [32] (Methods).

For Env(17/20) the carrier frequency of selecting HLA class I molecules (*f*_*H*_) was ≤77% but <40% for the other 4 polymorphisms considered (Table 1, column A rows 1–4). If we relax our definition of what KIR2DL2 can recognise and allow the variant amino acid on all peptide positions except position 2 and the C-terminal position, which are hidden in the HLA binding pockets, the frequency of selecting HLAs increases, reaching a median of 42% across all 6 amino acid polymorphisms (Table 1, column B, rows 1–4). KIR2DL2 mainly binds HLA-C1 group molecules and has a preference for 9mers [26]. As expected, if we only allow presentation by HLA-C1 group molecules or analyse binding of 9mers only, the frequency of selecting HLAs is significantly reduced (Table 1, columns C & D rows 1–4).

**Table 1:** Frequency of selecting HLA class I molecules in the US HIV+ population (*f*_*H*_). NK cells respond to (A) mutations at PC-1 (i.e. 1 residue from the C terminus) or PC-2 in peptides presented by any HLA-C, B*46:01 or B*73:01 molecule, (B) mutations at any position except residue 2 and PC, (C) mutations at PC-1 or PC-2 of 9mers only or (D) mutations at PC-1 or PC-2 in peptides presented by HLA-C1 group molecules only. HLA molecules are considered to be ‘selecting’ if they bind a variant peptide that obeys the definitions (A-D) above, or if they do not bind the wildtype peptide but bind at least one variant peptide; the latter figure is added to give the total frequency of selecting HLAs reported in the table below (see S1 Text for example calculation).

	Frequency of selecting HLAs (*f*_*H*_)			
Protein(position)	A	B	C	D
Env(17/20)	0.77	0.77	0.04	0.47
Vpu(71/74)	0.30	0.32	0.20	0.30
Gag(138)	0.001	0.02	0	0
Nef(9)	0.37	0.51	0.02	0.35
Tat(3)	0.11	0.11	0.11	0
Vpu(3)	0.17	0.40	0.17	0.17

Repeating the analysis with an alternative definition of epitope binding (based on the rank of a variant peptide relative to all other peptides of the HIV-1 proteome [33]; S1 Table and S1 Fig) and with alternative, independent epitope prediction software (Epipred; S2 Table) confirmed our finding that the frequency of selecting HLA class I molecules is low.

Next, we extended the analysis to the other viral polymorphisms associated with inhibitory KIR genes identified in [19]. As for the variants initially focussed on, the frequency of selecting HLAs for the additional polymorphisms was low (median 14% if we require the polymorphism at position PC-1 or PC-2, median 26% if we allow the polymorphism at any non-anchor position, Table 1, rows 5–6).

In summary, with the exception of Env (17/20), the proportion of the population where the variant has a selective advantage is low for all of the viral polymorphisms associated with the presence of inhibitory KIR genes in [19].

### Experimental confirmation of poor binding of variant peptide

Prediction of HLA class I-peptide binding using NetMHCpan is now highly accurate and the magnitude of the discrepancy between experiment and prediction is on a par with discrepancies between laboratories [34]; for HLA-C molecules the algorithm identifies ≥90% of epitopes at a false positive rate of ~2.5% (M. Nielsen pers. comm.) [35]. Nevertheless, we sought to experimentally confirm our finding that the majority of HLA C molecules do not stably bind peptides spanning the polymorphisms of interest using a peptide-MHC class I disassociation assay [36]. We focussed on one viral polymorphism (Gag 138L) and measured the stability of 8-, 9-, 10- and 11-mers containing this position at the terminal end (PC-1, PC-2 or PC-3) in complex with all HLA C molecules with an allele frequency ≥0.015. Seven of the ten HLA-C molecules tested showed no binding to any of the peptides (Table 2). Only HLA-C*07:01, HLA-C*07:02 and HLA-C*12:03 were able to bind a subset of the peptides and of these, only one HLA:peptide combination (HLA-C*12:03 –SQNYPIVQNLQ) had a half-life of more than an hour, the suggested minimum stability threshold for immunogenicity [37]. If we use the experimental measurements for frequent alleles and make the very generous assumption that all HLA molecules that were not tested bind the peptide then we find that the carrier frequency of selecting HLAs is *f*_*H*_ = 0.136. This estimate represents a maximum upper bound on the frequency of HLA class I molecules selecting for Gag138L.

**Table 2:** Stability of variant peptide:HLA-C complexes. The half-life (in hours) of the peptide: HLA complex is shown. A 0.00 indicates that the signal at time zero was less than 10% of the positive control and the half-life was too short to be measurable (the peptide is classified as non-binding).

	Peptide: HLA half-life (hours)									
Sequence	C*03:03	C*03:04	C*04:01	C*05:01	C*06:02	C*07:01	C*07:02	C*08:02	C*12:03	C*15:02
YPIVQNLQ	0.00	0.00	0.00	0.00	0.00	0.03	0.00	0.00	0.00	0.00
PIVQNLQG	0.00	0.00	0.00	0.00	0.00	0.00	0.00	0.00	0.00	0.00
NYPIVQNLQ	0.00	0.00	0.00	0.00	0.00	0.03	0.18	0.00	0.00	0.00
YPIVQNLQG	0.00	0.00	0.00	0.00	0.00	0.09	0.00	0.00	0.00	0.00
PIVQNLQGQ	0.00	0.00	0.00	0.00	0.00	0.06	0.00	0.00	0.00	0.00
YPIVQNLQGQ	0.00	0.00	0.00	0.00	0.00	0.04	0.15	0.00	0.00	0.00
NYPIVQNLQG	0.00	0.00	0.00	0.00	0.00	0.12	0.20	0.00	0.00	0.00
QNYPIVQNLQ	0.00	0.00	0.00	0.00	0.00	0.00	0.00	0.00	0.00	0.00
NYPIVQNLQGQ	0.00	0.00	0.00	0.00	0.00	0.03	0.14	0.00	0.00	0.00
QNYPIVQNLQG	0.00	0.00	0.00	0.00	0.00	0.04	0.00	0.00	0.00	0.00
SQNYPIVQNLQ	0.00	0.00	0.00	0.00	0.00	0.03	0.17	0.00	1.12	0.00

This experimental work, together with the comprehensive analysis using prediction algorithms, shows that the variant peptide fails to bind most HLA-C molecules. Consequently, the variant virus will only have a selective advantage in a small fraction of the total population.

### Effect of low proportion of selecting individuals on variant enrichment

**Fig 1:**
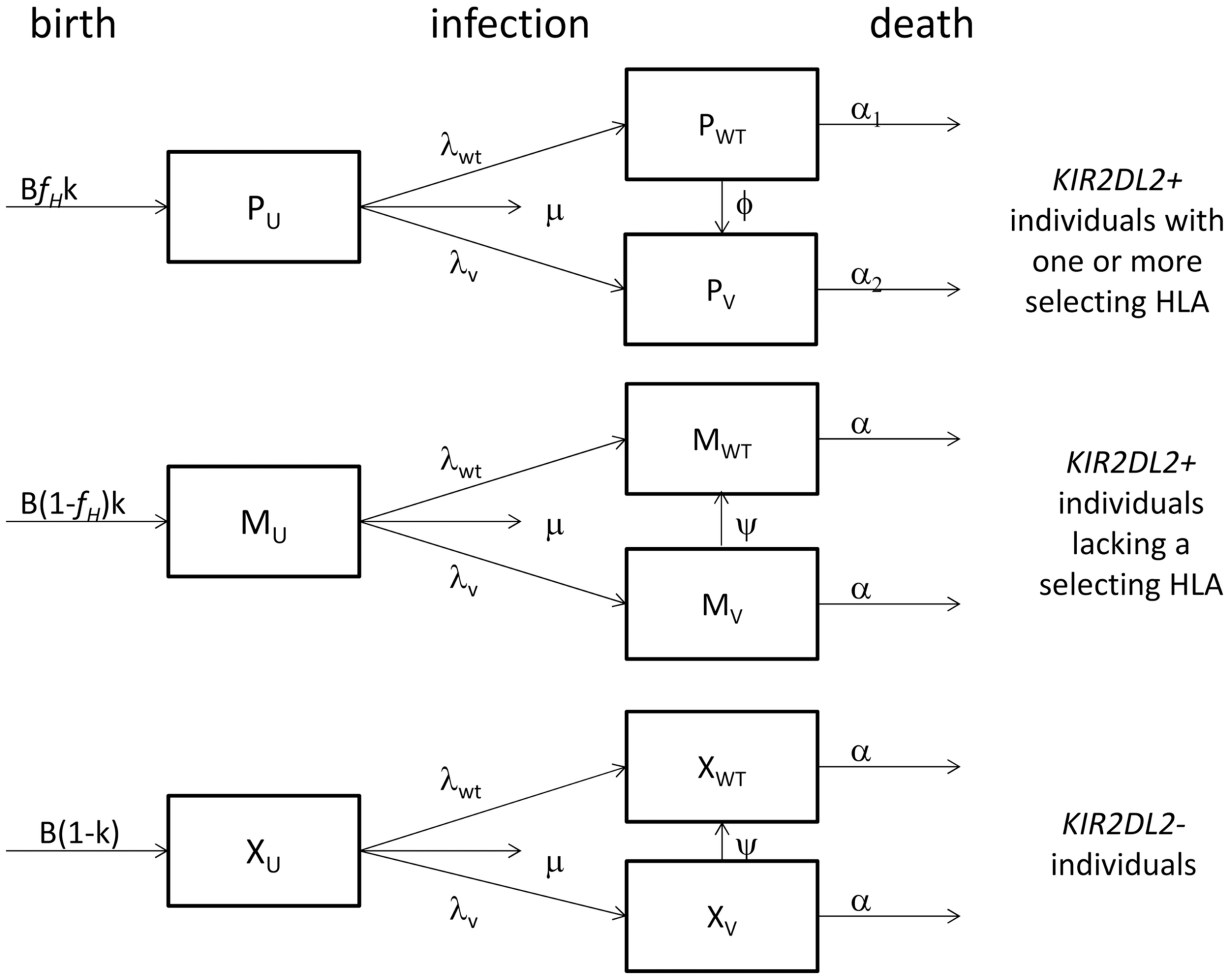
Schematic representation of the mathematical model used to describe viral evolution. We consider the dynamics of wildtype (WT) and variant (V) virus infection in a population of *KIR2DL2*^**+**^ selectors (P), *KIR2DL2*^**+**^ non-selectors (M) and *KIR2DL2*^**–**^ individuals (X). HIV-1-negative individuals (subscript U), are born at rate B and can become infected with either WT or V (λ_WT_ and λ_V_ respectively), at a rate depending on the prevalence of each strain in the total population. The fraction of *KIR2DL2*^**+**^ people in the total population is *k* and the frequency of selecting HLAs is *f*_*H*_. In *KIR2DL2*^**+**^ selectors, virus can escape from WT to V, in the other two groups V strains can revert to WT. Uninfected individuals die at a rate μ and HIV-1-infected individuals die at an increased rate α.

We next asked whether these low proportions of selecting individuals are sufficient to drive the enrichment of variant virus in *KIR2DL2*^+^ individuals reported by Alter *et al*. We constructed a mathematical model that simulates wild type and variant HIV-1 infection. We considered the *KIR2DL2*^+^ selector population, where the variant has an advantage and escape can occur, and the *KIR2DL2*^+^ non-selector and *KIR2DL2*^–^ population, where variant virus does not have an advantage so escape will not occur and there is the possibility of reversion from variant to wild type virus. A schematic of the model is given in Fig 1, see Methods for details.

**Fig 2:**
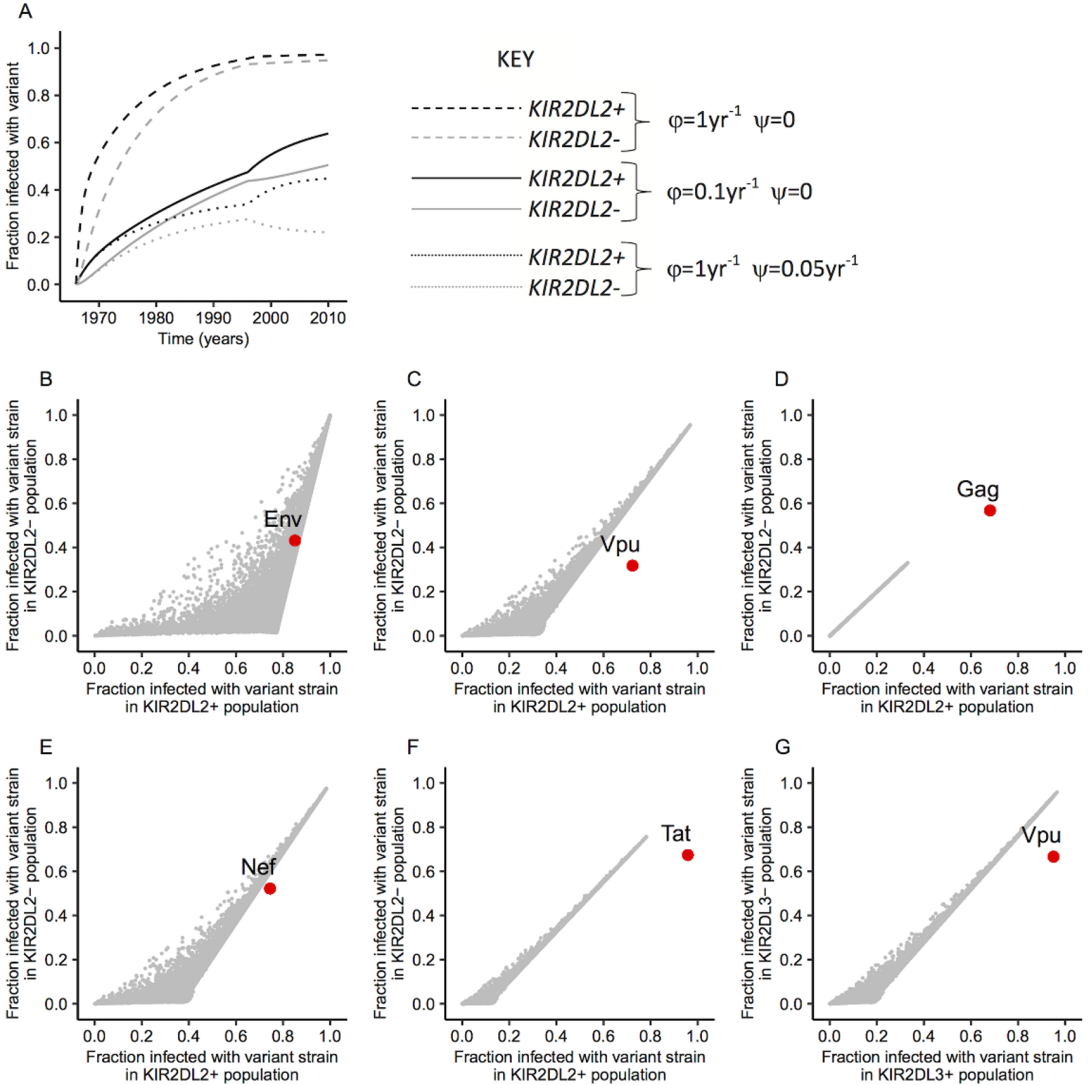
Fraction of the population infected with variant virus. (**A)** time course of the fraction of Env-variant-infected individuals in the *KIR2DL2*^**+**^ (black lines) and *KIR2DL2*^**–**^ (grey lines) population from the start of the epidemic for three different combinations of escape (φ) and reversion (ψ) rates (solid: φ = 0.1yr^-1^ and ψ = 0; dashed: φ = 1yr^-1^ and ψ = 0; dotted: φ = 0.1yr^-1^ and ψ = 0.05yr^-1^). The abrupt change in the late 1990s is due to the introduction of combination antiretroviral therapy. Only 3 combinations of escape and reversion shown for ease of visualisation; a total of 100,000 combinations were considered **(B-F)** Fraction of variant-infected individuals in the *KIR2DL2*^**+**^ and *KIR2DL2*^**–**^ population for 100,000 randomly chosen parameter values. Grey dots: model predictions, red circle: observation reported by Alter *et al*. **B**: Env, **C**: Vpu, **D**: Gag, **E**: Nef, **F**: Tat. Model parameters are varied within the ranges given in S3 Table. **(G)** Predicted and observed variant enrichment for the *KIR2DL3*-associated polymorphism in Vpu (3).

We studied the increase of variant virus in the population over time with parameter values in the centre of the physiological ranges (S3 Table). Assuming an escape rate of 0.1 yr^-1^ and no reversion, the model predicts a steady increase in variant virus in both the *KIR2DL2*^+^ and *KIR2DL2*^–^ population (Fig 2A, solid line). If we increase the escape rate, the fraction of variant infected people increases faster in both populations (Fig 2A, dashed line); if we increase the reversion rate, the fraction of variant infected people increases more slowly in both populations (Fig 2A, dotted line). We find that the fraction of variant-infected people in the modelled *KIR2DL2*^–^ population is very similar to that in the *KIR2DL2*^+^ population, while in the observed population there was a significant enrichment of the variant in *KIR2DL2*^*+*^ people. The abrupt change in the modelled dynamics of variant enrichment in the late 1990s is due to the introduction of combination antiretroviral therapy. Lifespan is extended giving the variant longer to emerge in selectors and longer to revert in non-selectors.

To investigate if, despite the low frequency of selecting HLAs, any combination of parameters could predict the experimental data we simulated the population dynamics for 100,000 random parameter sets taken from realistic ranges for the HIV-1-epidemic in the USA. We then predicted the enrichment of the polymorphism in the *KIR2DL2*^*+*^ (Fig 2B–2F), and *KIR2DL3*^*+*^ (Fig 2G) populations in 2010 using the frequency of selecting HLAs (*f*_*H*_) for each polymorphism (Table 1) and compared this with the experimental data. It is clear that, with the exception of one of the variants (Env 17/20) out of six, none of the parameter combinations considered can predict the enrichment of variant virus. Relaxation of the definition of KIR binding (S2 Fig) as well as variation of the exact definition of variant polymorphism (S2 Text Additional analysis) confirmed this result.

These results suggest that the enrichment of viral polymorphisms associated with inhibitory KIR genes cannot be explained by KIR binding of HLA-C molecules presenting variant epitopes.

### Effect of T cell selection pressure

**Fig 3:**
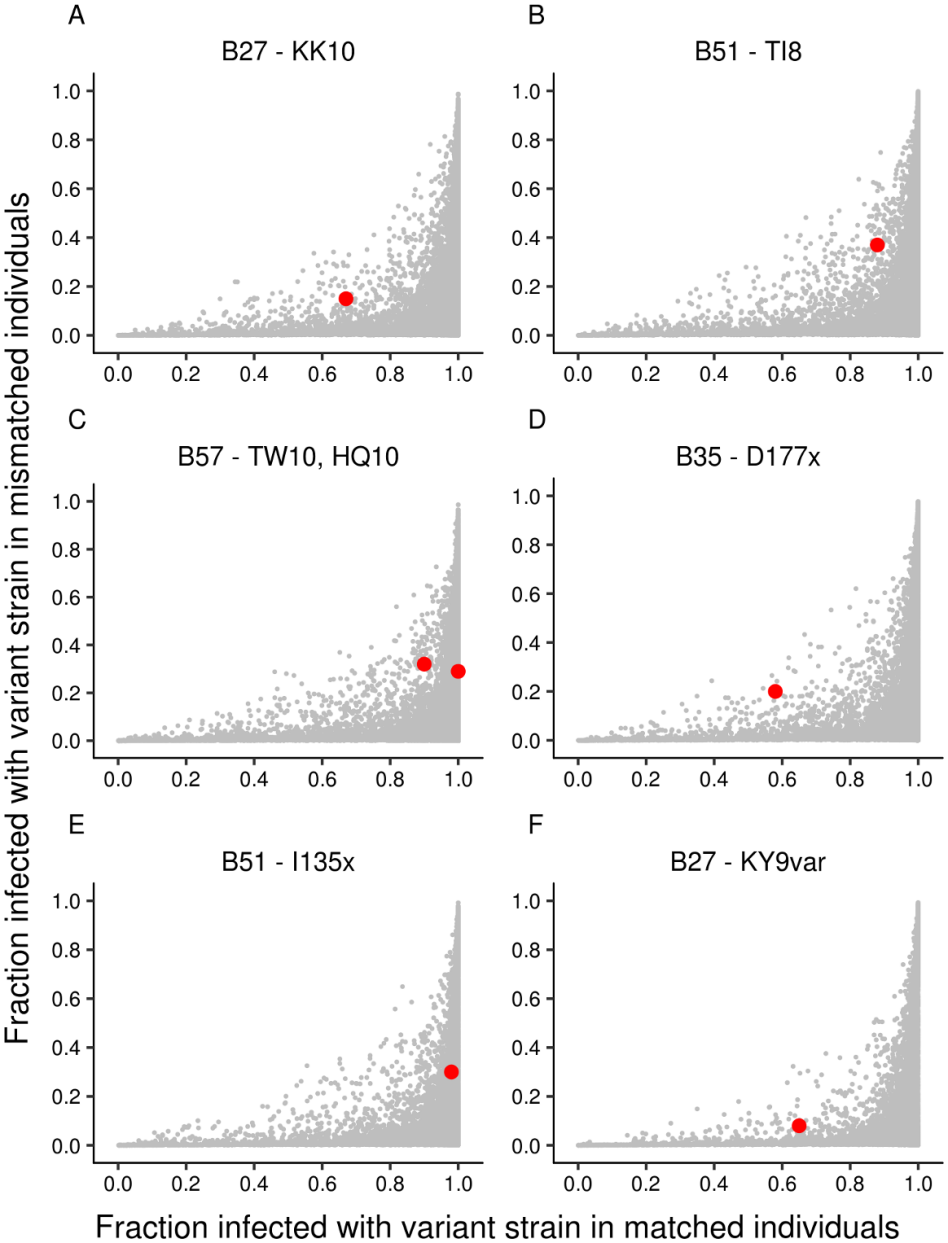
Model predictions for enrichment of CD8^+^ T cell escape variants. In contrast to the enrichment of NK cell escape variants in *KIR2DL2*^*+*^ individuals, the enrichment of CD8^+^ T cell escape variants in HLA-matched individuals is readily predicted. The 6 panels relate to 7 different reported enrichments of viral polymorphisms. Variant enriched in individuals carrying **(A)** HLA-B*27+ [38], **(B)** HLA-B*51+ [38] **(C)** HLA-B*57+ [38] **(D)** HLA-B*35:*01+* [40] **(E)** HLA-B*51:*01+* [40] **(F)** HLA-B*27+ [39]. Grey dots: model predictions, red circle: experimental data.

As a positive control, we investigated whether our approach can predict enrichment of CD8^+^ T cell escape mutations in HLA-matched compared with HLA-mismatched populations. We considered seven polymorphisms from three published studies [38–40]. We found that the model could successfully predict enrichment of HLA-associated polymorphisms in all seven cases (Fig 3). NK cells and T cells differ in many ways. However, the aspect that we are investigating: requirement for presentation of viral peptide by HLA class I molecules, is shared. This comparison is therefore an appropriate control.

### Frequency of selecting HLAs required to explain the observed enrichment of variant virus in *KIR2DL2*^+^ population

We next investigated if increasing the frequency of selecting HLAs (*f*_*H*_) could enable us to predict the experimentally observed enrichment of variant virus in *KIR2DL2+* individuals (S3 Fig). In all cases the enrichment could be predicted provided that the frequency of selecting HLAs was considerably higher. We found that the enrichment of Gag 138L can be predicted with *f*_*H*_ = 25–35%, enrichment of Nef 9S with *f*_*H*_ = 50–75%, and enrichment of Vpu 71M/74H-Env 17W/20M and Tat 3S with *f*_*H*_ = 80–100%.

### Impact of polymorphisms on peptide processing

Polymorphisms in neighbouring sequences can alter the processing of the peptides they flank [41]. So another possible explanation for the advantage conferred by a variant is that the mutation increases the production of nearby binding peptides and thus indirectly affects the level of HLA class I:peptides available for KIR binding. To test this possibility, we used NetMHCpan to predict the binding of peptides within 20 amino acids of the six polymorphisms (Env(17/20), Vpu(71/74), Gag(138) and Nef(9)) to HLA-C alleles, HLA-B*73:01 and HLA-B*46:01 and found 38 flanking binders. We then used NetCTLPan v1.1 to predict the ability of these peptides to be cleaved and transported with both the variant and the wildtype flanking region. We found that cleavage was only increased in the presence of the variant for one peptide that binds HLA-C*15:08. HLA-C*15:08 has not been reported in African American, white or Hispanic populations (which together constitute the vast majority of HIV-1-infected individuals in the US). We conclude that the impact of the polymorphism on flanking peptide processing is unlikely to contribute to the fraction of selectors in the population.

### Polymorphisms associated with activating KIRs

Alter *et al* also report 14 polymorphism enrichments associated with activating KIRs (aKIRs) but do not study these variants further (Table 1 in [19]). We extended our analysis to calculate the frequency of selecting HLAs associated with each of these polymorphisms. An HLA class I molecule was considered to be selecting if it ligates the activating KIR and either the HLA molecule binds the wild type but not the variant peptide (variant decreases NK cell activation by decreasing ligand availability) or the HLA molecule binds at least one wildtype peptide with the polymorphic position at PC-1 or PC-2 (later extended to any non-anchor position) (i.e. variant decreases NK cell activation by altering aKIR signalling). As for the inhibitory KIRs, *f*_*H*_ was very low with the exception of one *KIR2DS3*-associated polymorphism at Vpr37 (S4 Table), indicating that, in the majority of cases the variant strain has a selective advantage in a small proportion of the population.

### Model-independent approach

**Fig 4:**
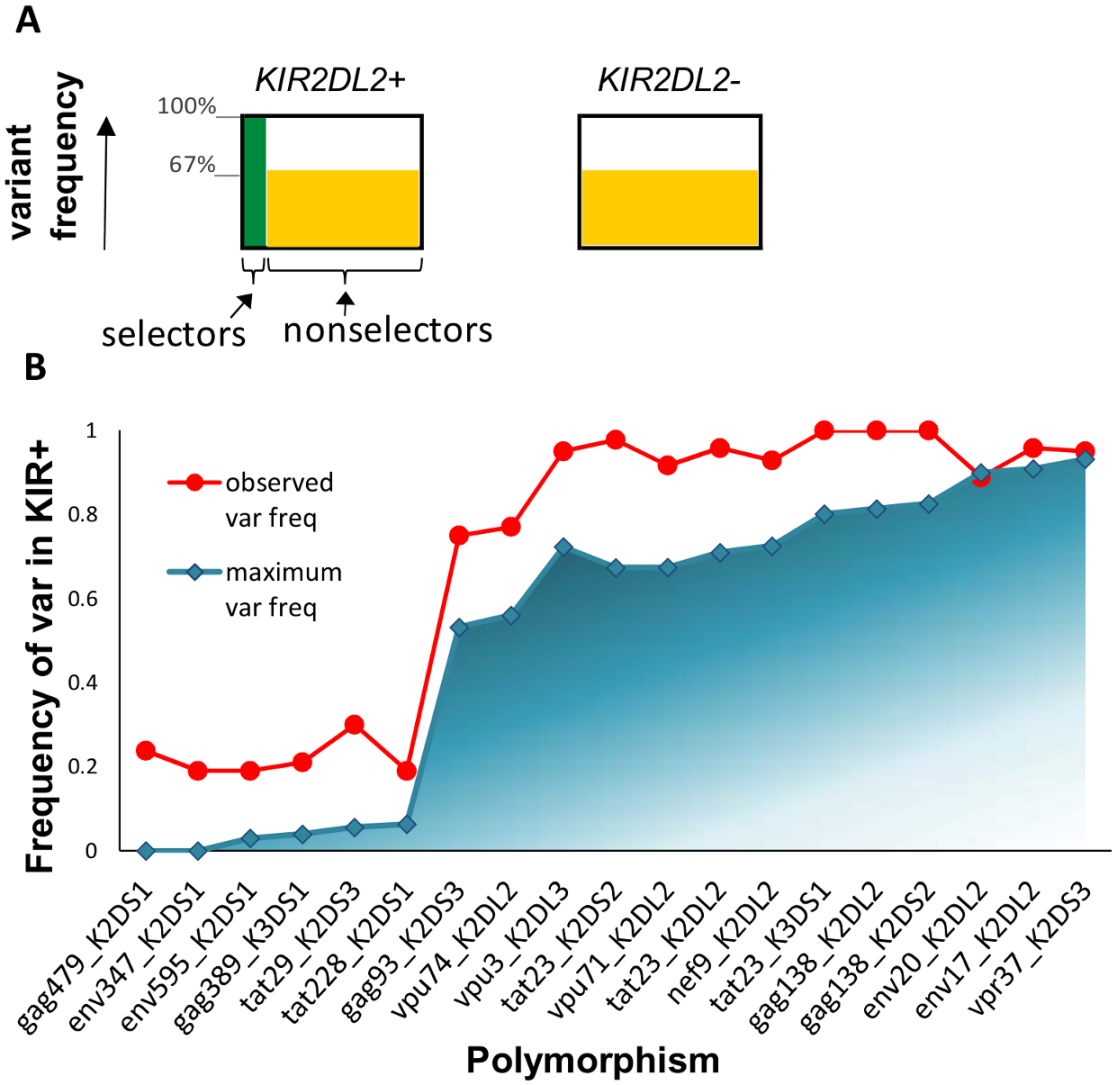
Model-independent approach. **(A) Schematic.** Under the hypothesis of Alter *et al* the frequency of the variant in *KIR2DL2+* non-selectors and *KIR2DL2-* non-selectors will be similar (orange; 67% for the example of Tat(3)). Even if the frequency of the variant in *KIR2DL2+* selectors is 100% (green) it is trivial to see that, because of the low frequency of selectors amongst the *KIR2DL2+* population, the variant frequency amongst the whole *KIR2DL2+* population is low and is similar to that of the *KIR2DL2-* population. **(B) Results.** The maximum variant frequency expected (blue) is systematically lower than the observed variant frequency (red) for the majority of the polymorphisms described by Alter *et al*. The exceptions are Env 17/20:*KIR2DL2* and Vpr 37:*KIR2DS3* already discussed. Shading indicates that the expected variant frequency can be lower (but not higher) than the maximum variant frequency, i.e. variant frequency can take any point in the shaded region depending on the variant frequency in selectors, the maximum is attained when variant frequency in selectors is 100% (solid blue line).

To check that our conclusions (that the observed variant enrichments were incompatible with the low proportion of selectors) were independent of the model assumptions we performed a simple, “model-independent” calculation of the maximum variant enrichment attainable. As a concrete example consider Tat(3); Fig 4A. The polymorphism at this position is present in 96% of *KIR2DL2+* individuals and 67% of *KIR2DL2-* individuals; the frequency of selecting HLAs (*f*_*H*_) is 11%. Under the hypothesis of Alter *et al*, the selection pressure at this position will be similar in all non-selectors (*KIR2DL2*^*+*^ individuals without the selecting HLA and all *KIR2DL2*^-^ individuals); and will be determined by the fitness of the variant in these non-selecting hosts. The variant frequency in *KIR2DL2*^-^ and *KIR2DL2*^*+*^ non-selectors will therefore be approximately equal at the observed value of 67%. The variant frequency in all *KIR2DL2*^*+*^ individuals is then

$$\begin{array}{ll} \begin{array}{c} \text{Variant freq in}\\ KIR2DL2^{+}\\ \text{individuals} \end{array} & =[\begin{array}{ccc} \text{Freq of} & & \text{Variant freq}\\ KIR2DL2^{+} & \times & \text{in }KIR2DL2^{+}\\ \text{selectors} & & \text{selectors} \end{array}]+\left[\begin{array}{ccc} \text{Freq of} & & \text{Variant freq}\\ KIR2DL2^{+} & \times & \text{in }KIR2DL2^{+}\\ \text{non-selectors} & & \text{non-selectors} \end{array}\right]\\ & =[\begin{array}{ccc} \text{ 0.11 } & \times & v\\ & & \; \end{array}]+\left[\begin{array}{ccc} \left(1-0.11\right) & \times & 0.67\\ \; & & \; \end{array}\right] \end{array}$$

where *v* is the variant frequency in *KIR2DL2*^*+*^ selectors. It can readily be seen that even in the extreme case of 100% frequency of the variant in *KIR2DL2*^*+*^ selectors (*v* = 1) the maximum variant frequency that can be attained across all *KIR2DL2*^*+*^ individuals is 70.6%, considerably lower than the observed frequency (96%). Repeating this across all polymorphisms shows a clear pattern (Fig 4B). All observed variant frequencies (with the exception of the Env:*KIR2DL2* and Vpu:*KIR2DS3* polymorphisms already discussed), are systematically higher than the maximum expected based on the hypothesis of Alter *et al* (*P* = 8x10^-6^). It is striking that the maximum variant frequency is strongly positively correlated with the observed variant frequency (*P* = 2x10^-12^). This is not simply because the maximum variant frequency is correlated with the variant frequency in non-selectors, or with the frequency of selectors since, in a multivariate linear regression, all 3 variables are independent predictors of the maximum variant frequency (observed frequency in selectors *P = 0*.*009*, observed frequency in non-selectors *P = 2x10*^*-6*^, f_H_ *P = 4x10*^*-10*^).

## Discussion

NK cell inhibition or activation via KIRs is determined by HLA class I ligands that bind the KIR receptors and, to a lesser extent, by the sequence of the peptides presented by these ligands [2–8]. Under the hypothesis of Alter *et al* [19–22], a necessary (but not sufficient) condition for a host to exert KIR-mediated selection pressure for a given variant is possession of an HLA class I molecule that ligates the KIR in question and presents the variant peptide. We calculated the proportion of hosts meeting this condition and investigated whether it was consistent with the enrichment of variant virus previously reported [19]. We found that the proportion of the population that can select for the variant is low and is insufficient to explain the observed enrichment of the variant polymorphism in the *KIR2DL2*^+^-population for all but one of the 6 polymorphisms focussed on by Alter *et al* [19]. Extension to the other 13 polymorphisms associated with inhibitory and activating KIR genes showed identical behaviour. A simple, “model-independent” approach confirmed this finding. The problem is very simple: the frequency of HLA class I molecules that bind a given KIR and that are capable of exerting selection pressure at the same amino acid position is too low to explain the variant enrichment seen in the population. This is not to say that selection pressure is low, but that in different people with different HLA molecules, selection pressure will be exerted at different points in the virus genome. So, on studying virus sequence in the whole population, a signal would not be detected since any given amino acid would only be under selection pressure in a minority of individuals. Furthermore, not all individuals who have the potential to select for an NK escape variant will do so, further reducing the variant enrichment. In order to predict the observed variant enrichment the proportion of selectors needs to be considerably higher.

KIR-HLA:peptide ligation requires two steps: binding of the peptide to the HLA molecule and ligation of the HLA:peptide complex by KIR. Here we focus on HLA:peptide binding, a necessary but not sufficient step for HLA:peptide-KIR binding. HLA-peptide binding is well-characterised and tractable both with experimental and *in silico* techniques; less is known about KIR ligation by the HLA-peptide complex. We therefore assume that all HLA:peptide complexes will trigger KIR. This will overestimate the fraction of selectors. The finding that we cannot predict the observed variant enrichment even with this overestimated proportion of selectors underscores the discrepancy between the hypothesis of Alter *et al* and their observed experimental data.

Our estimate of the proportion of the population that could select for variant virus depends on binding prediction algorithms. The algorithm that we use (NetMHCpan v2.8) is a highly accurate quantitative predictor, with an error comparable to experimental error [32, 34, 42, 43]. We also predict HLA-peptide binding using an independent prediction algorithm, Epipred. Epipred and NetMHCpan use different methods (logistic regression and artificial neural networks respectively) and are trained on different experimental data sets. Both these methods predict poor binding of variant peptides to the HLA ligands of the KIRs of interest. Additionally, poor binding of a subset of 11 variant peptides to 10 HLA-C molecules (total of 110 combinations) was confirmed experimentally using a peptide:MHC class I dissociation assay. Of note, two assumptions we made in estimating the fraction of selecting HLAs (that gene frequencies add linearly and that all amino acid changes will enhance KIR signalling) will both err on the side of caution and inflate the frequency of selecting HLAs. In reality, the true frequency will tend to be less than this estimate. We also explored the possibility of variants enhancing the cleavage of flanking epitopes compared to the wildtype but found that this could not explain the observed enrichment either.

In summary, we show that, of the 22 viral polymorphism enrichments associated with KIR genes listed in [19], 19 could be analysed; of these a total of 16 could not be explained by the hypothesis of Alter *et al*. A minority (3/19) could be explained, consistent with isolated examples of NK cell escape reported in the literature e.g. [22]. How else could the KIR-associated enrichment be explained for the remaining 16 polymorphisms? For concreteness, consider the example of the *KIR2DL2*-associated polymorphisms, similar arguments follow for the other KIRs. Variant enrichment in *KIR2DL2*^*+*^ individuals implies either that the enrichment is driven by a gene in linkage with *KIR2DL2* rather than *KIR2DL2* itself or that KIR2DL2 binds far more widely than has previously been appreciated or that variant peptides are indirectly impacting KIR ligation. We consider each of these possibilities in turn. *KIR2DL2* is in strong positive linkage with *KIR2DS2*, an activating KIR gene. Alter *et al* investigate the possibility that KIR2DS2 rather than KIR2DL2 is the selecting molecule but discount it because of greater variant enrichment in *KIR2DL2*+ compared with *KIR2DS2*+ individuals. However, due to the extremely tight linkage between the genes and the size of their cohort (N = 91) it is difficult to make this statement with confidence. However, KIR2DS2 fails to explain the data for exactly the same reason that KIR2DL2 does; the fraction of people with ligating HLA that also bind the wild type peptide is very low. *KIR2DL2* is also in linkage with other KIR genes. We considered whether KIR2DL2-related variants could also exert a selective advantage via KIR2DL1 or KIR2DL3, depending on the HLA-C restriction of the variant peptide. This would theoretically give rise to a group of *KIR2DL2*- individuals who would nevertheless also select for the escape variant. Under such an assumption, the proportion of both *KIR2DL2*+ and *KIR2DL2*- variant carriers would be higher, which would result in a less-pronounced enrichment in *KIR2DL2*^*+*^ individuals; again failing to predict the data. Looking beyond the KIR receptor complex the next gene cluster is the human leukocyte immunoglobulin-like receptor (LILR) family [44]. The LILR have been linked with outcome in HIV-1 [45], show peptide specificity and bind HLA molecules more broadly than KIR [44]. They are therefore an ideal candidate to explain the observed selection, which was attributed to KIR2DL2. However, although the LILR are separated from the KIR by only 450 kb, linkage between them is weak [46] and is probably insufficient to drive the observed variant enrichment in *KIR2DL2+* individuals. Furthermore, although Alter *et al* do not investigate the molecular mechanism underlying the NK cell escape they describe, they do present some limited functional work showing that a KIR2DL2-IgG fusion construct binds more strongly to Env-variant infected cells compared with wild type implying a direct role for KIR2DL2 rather than a genetically linked molecule. If linkage does not explain the variant enrichment, an alternative hypothesis is that KIR2DL2 binds more widely than is currently realised. We considered the possibility that KIR2DL2 could bind to peptide:HLA complexes which were bound with very low affinity. Nevertheless, there is a limit to how low the affinity of peptide for HLA can be as affinity and stability are closely related and the complex needs to be sufficiently stable to be presented on the cell surface. We have already considered peptides that bind with affinity as low as 500nM or rank<2% but still found an insufficient fraction of selectors. Recently, it was reported that a considerable fraction of self-peptides presented on HLA are spliced [47]. The mechanism behind this splicing has not yet been elucidated and it is not known whether spliced viral peptides are also presented. If spliced HIV-1 peptides are presented this could potentially increase the number of selectors. Another explanation is that the virus variants indirectly affect KIR-HLA binding by affecting HLA C expression levels. It has been shown that different HIV-1 strains downregulate surface HLA-C expression differentially [48]; related to this it is notable that all of the inhibitory KIR footprints identified by Alter and co-authors relate to lineage III KIRs which bind HLA C molecules rather than lineage II KIRs which bind HLA-A and -B. However, it is difficult to understand how many different polymorphisms in multiple proteins could all be implicated in HLA C downmodulation.

Our estimates of the fraction of selectors and epidemiological model to predict variant enrichment accurately reproduced published works on CD8^+^ T cell-mediated immune pressure on HIV-1; since the aspect of NK cell selection which we are modelling (requirement for presentation of viral peptide by HLA class I molecules) is shared by both NK cells and CD8^+^ T cells, this is an appropriate control. The conclusion, that our model of the hypothesis of Alter *et al* cannot predict the KIR-associated variant enrichment, demonstrates that an aspect of the KIR-HIV-1 interaction outside their hypothesis is involved. We suggest that, if this additional aspect were included in the model, then the model would successfully predict the observed variant enrichment. It is important to stress that we are not investigating the role of NK cells in controlling HIV-1 infection in general. There are a large number of studies convincingly demonstrating that NK cells are protective in HIV-1 [9–17]. We are focusing on whether this would result in selection that would be detectable at the population level.

We conclude that whilst Alter *et al*’s data is consistent with selection pressure, the postulated hypothesis is insufficient to explain the data. This forces a re-evaluation of the evidence that NK cells exert selection pressure in HIV-1 [19] and excitingly, suggests that there is a significant aspect of KIR immunobiology that we do not understand.

## Methods

### Prediction of HLA:Peptide binding

The binding of variant and wild type peptides from HIV-1 proteins to HLA class I molecules was calculated using NetMHCpan v2.8 [34] (S4 Fig). We considered a peptide to be a binder if its predicted binding falls within the top two percent of a set of 200,000 random natural peptides or has affinity <500nM. We repeated the analysis using an alternative definition of a binder, namely a peptide is considered to be a binder if its affinity lies within the top 10% of HIV-1-derived peptides [33]. Calculations were also repeated using independent prediction software Epipred [49]. Epipred and NetMHCpan use different methods (logistic regression and artificial neural networks respectively) and are trained on different experimental data sets.

### Experimental measurement of peptide-HLA-I complex stability

The stability of 8-, 9-, 10- and 11-mers containing Gag 138L at the terminal end (PC-1, PC-2 or PC-3) in complex with HLA molecules with an allele frequency ≥0.015 in the HIV-1-infected population was measured as previously described [36]. Briefly, recombinant, denatured and biotinylated MHC-I alpha chain (50-200nM) was diluted in PBS/0.1% Lutrol F68 containing 10μM of peptide and trace amounts of 125I radiolabeled β2m in 384 well streptavidin coated scintillation microplates (Streptavidin FlashPlate HTS PLUS SMP410001PK, Perkin Elmer). Flashplates were incubated over night to attain peptide-HLA-I complex folding. Peptide-HLA-I complex dissociation was initiated by adding excess of unlabeled β2m (200nM) and transferring the plate to a TopCount NXT Liquid Scintillation Reader (Perkin Elmer) at 37°C. The plate was read continuously for 24 hours. The peptide off-rate was calculated according to an exponential decay equation: Y = Y0*e^(-k*x), where x is the time in hours and k is the off-rate (complex half-lives were calculated by T½ = ln(2)/k). Relevant positive controls were included for each HLA-I molecule. Peptides were categorised as not binding if the signal intensity at time point 0 was less than 10% of the relevant positive control.

### Determination of selecting HLA molecules

An HLA molecule was said to be capable of selecting for an inhibitory KIR-associated variant if 1) the HLA molecule ligates the relevant iKIR and 2i) the variant peptide was predicted to bind the HLA molecule and had a polymorphism in a position that would interact with the KIR receptor (initially considered to be PC-1 or PC-2, later relaxed to any non-anchor position) or 2ii) the HLA molecule binds none of the wild type peptides and at least one of the variant peptides containing the variant position. An HLA class I molecule was considered to be capable of selecting for an activating KIR-associated variant if 1) the HLA molecule ligates the relevant aKIR and either 2i) the HLA molecule binds at least one wild type peptide with the polymorphic position at PC-1 or PC-2 (later extended to any non-anchor position) (i.e. variant decreases NK cell activation by altering aKIR signalling) or 2ii) the HLA molecule binds the wild type but not the variant peptide (variant decreases NK cell activation by decreasing ligand availability).

Since we were unable to reliably predict whether an amino acid change enhanced KIR signalling we made the generous assumption that any change at PC-1 (i.e. 1 residue from the C terminus) or PC-2 (later relaxed to any change at any non-anchor position) will enhance signalling. This assumption will lead to an overestimate of *f*_*H*_, i.e. we are erring on the side of caution. See S1 Text for an example of selecting HLA molecules.

### Calculation of the frequency of selecting HLA molecules in the US HIV-1-infected population

The frequency of selecting HLA molecules (*f*_*H*_) for a given KIR-associated polymorphism is estimated by first add up the gene frequency of selecting HLA alleles in each ethnic group (this will double count people who carry more than one selecting allele and will thus overestimate *f*_*H*_ erring on the side of caution). We then determined the population frequency of selecting HLAs for each ethnic group using:

$$f_{H_{i}}=x_{i}\left(1-x_{i}\right)+\left(1-x_{i}\right)x_{i}+x_{i}^{2}$$

Where $f_{H_{i}}$ is the carrier frequency of selecting HLAs in ethnic group *i* and *x*_*i*_ is the frequency of genes coding for selecting HLA class I molecules in that ethnic group [30]. Next, we multiplied the carrier frequency of selecting HLAs in one ethnic group with the frequency of the group in the HIV-1-infected population [50] and finally summed over all ethnic groups to give the carrier frequency of selecting HLAs in the HIV-1-infected population (*f*_*H*_)

$$f_{H}=\sum_{i}f_{H_{i}}.freq(i)$$

### Model of viral evolution

We used a model of viral evolution based on [51]. For the sake of clarity we describe the model for studying polymorphisms enriched in the *KIR2DL2*^*+*^ population; the same model can be generalised to any KIR of interest. We describe 9 populations of individuals: individuals who are *KIR2DL2*^*+*^ and carry one or more selecting HLA who are uninfected (P_U_), wild type (WT)-infected (P_WT_) or variant (V)-infected (P_V_), *KIR2DL2*^+^ without selecting HLA who are uninfected (M_U_), WT-infected (M_WT_) or V-infected (M_V_) and *KIR2DL2*^–^ uninfected (X_U_), WT-infected (X_WT_) or V-infected (X_V_). NK cells can drive escape in *KIR2DL2*^+^ individuals with selecting HLA (P_WT_), reversion can occur in V-infected *KIR2DL2*^+^ hosts without selecting HLA and in *KIR2DL2*^–^ individuals (M_V_ and X_V_). The model is described by 9 ordinary differential equations:

$$\begin{array}{ll} {\dot{P}}_{U} & =f_{H}kB-\left(\lambda_{WT}+\lambda_{V}+\mu \right)P_{U}\\ {\dot{P}}_{WT} & =\lambda_{WT}P_{U}-\phi P_{WT}-\alpha_{1}^{}P_{WT}\\ {\dot{P}}_{V} & =\lambda_{V}P_{U}+\phi P_{WT}-\alpha_{2}P_{V}\\ {\dot{M}}_{U} & =\left(1-f_{H}\right)kB-\left(\lambda_{WT}+\lambda_{V}+\mu \right)M_{U}\\ {\dot{M}}_{WT} & =\lambda_{WT}M_{U}+\psi M_{V}-\alpha M_{WT}\\ {\dot{M}}_{V} & =\lambda_{V}M_{U}-\psi M_{V}-\alpha M_{V}\\ {\dot{X}}_{U} & =\left(1-k\right)B-\left(\lambda_{WT}+\lambda_{V}+\mu \right)X_{U}\\ {\dot{X}}_{WT} & =\lambda_{WT}X_{U}+\psi X_{V}-\alpha X_{WT}\\ {\dot{X}}_{V} & =\lambda_{V}X_{U}-\psi X_{V}-\alpha X_{V} \end{array}$$

where *f*_*H*_ is the carrier frequency of selecting HLA class I molecules in the HIV-1-infected US population, *k* is the fraction of *KIR2DL2*^+^ individuals in the US population, *B* is the birth rate in the US, μ is the death rate of uninfected individuals, α, α_1_, α_2_ are the death rates of HIV-1-infected individuals (see below for details), **φ** is the escape rate of WT virus to V and ψ is the reversion rate from V to WT. Infection rates λ_WT_ and λ_V_ are defined as:

$$\lambda_{WT}=\frac{\beta }{N}\left(P_{WT}+M_{WT}+X_{WT}\right)$$

$$\lambda_{V}=\frac{\beta }{N}\left(P_{V}+M_{V}+X_{V}\right)$$

where β is transmission probability and N is the size of the total population. Simulations are run from the time of first infection in the US (t0) until 2010. At the introduction of combination antiretroviral therapy (cART) in 1996, the death rate of HIV+ individuals (α) and transmission probability (β) change. The impact on mortality of HIV-1 escape from the NK cell response is unknown. Two schemes were considered:

1. A selector with escape variant virus has a mortality disadvantage compared to all other individuals (α_1_ = α <α_2_).
2. A selector with wild type virus has a mortality advantage compared to all other categories of HIV-1-infected individuals (α_1_<α_2_ = α).

The fraction of V infected individuals in the *KIR2DL2*^+^ population is calculated as:

$$f=\frac{P_{V}+M_{V}}{P_{V}+M_{V}+P_{WT}+M_{WT}}$$

To investigate impact of parameter choice, the model was run for 100,000 random parameter sets taken from realistic parameter ranges for the HIV-1-epidemic in the USA.

Parameter values are given in S3 Table, a schematic of the model is presented in Fig 1.

### Model-independent estimate of maximum variant enrichment

The variant enrichment in a *KIR+* population will depend on the variant enrichment in selectors and non-selectors in that population vis

$$\begin{array}{ll} \begin{array}{c} \text{Variant freq in}\\ KIR^{+}\\ \text{individuals} \end{array}= & \left[\begin{array}{ccc} \text{Freq of} & & \text{Variant freq}\\ KIR^{+} & \times & \text{in }KIR^{+}\\ \text{selectors} & & \text{selectors} \end{array}\right] \end{array}+\left[\begin{array}{ccc} \text{Freq of} & & \text{Variant freq}\\ KIR^{+} & \times & \text{in }KIR^{+}\\ \text{non-selectors} & & \text{non-selectors} \end{array}\right]$$

The frequency of *KIR+* selectors and non-selectors is calculated as described above (“Calculation of the proportion of selectors in the US HIV-1-infected population”). The variant frequency in *KIR+* non-selectors will be similar to *KIR-* non-selectors and has been measured experimentally and reported in Alter *et al*. We therefore know 3 of the 4 terms on the right hand side of the equation above, the only unknown is “variant frequency in *KIR+* selectors”. The maximum variant enrichment in *KIR+* individuals will be attained when the “variant frequency in *KIR+* selectors” is maximised i.e. 1.

## Supporting information

S1 Text Selecting HLA molecules: A worked example. (DOCX)

S2 Text Supplementary analysis. (DOCX)

S1 Table Fraction of selecting HLAs based on rank definition of an epitope (NetMHCpan). An alternative definition of whether a peptide binds an HLA molecule is the rank of its binding affinity compared to the binding affinity of all peptides from the viral proteome. The idea is that only viral peptides in the top 5 or 10 binders for a single HLA molecule will be competitive [33]. Table below shows the median and minimum rank of the variant peptide with the highest affinity relative to the whole HIV-1 genome, the number of HLA alleles for which the highest ranking peptide falls into the top 5 or top 10 of the whole HIV-genome and the carrier frequency of selecting HLAs (*f*_*H*_) based on this definition.
(DOCX)

S2 Table Fraction of selecting HLAs based on rank definition of an epitope (Epipred). The carrier frequency of selecting HLAs (*f*_*H*_) was calculated using an alternative, independent epitope prediction algorithm Epipred. Results are tabulated below.
(DOCX)

S3 Table Parameter values for the model of viral evolution. (DOCX)

S4 Table Frequency of selecting HLA’s for polymorphisms associated with activating KIR. HLA ligands of many of the activating KIRS are unknown and/or contentious. We therefore considered all commonly assumed ligands (column “assumed HLA ligand”). KIR2DS5 is believed to have evolved from an activating C2 receptor but appears to have lost the capacity to bind HLA class I [52]; the two polymorphisms identified by Alter *et al* as associated with KIR2DS5 were therefore excluded from our analysis. Additionally, a third polymorphism identified by Alter *et al* (Env 46 KIR3DS1) was excluded as we were unable to identify the variant amino acid.
(DOCX)

S1 Fig Rank of binding affinity for peptides containing amino acid polymorphisms. How strongly a peptide binds an HLA molecule relative to other peptides of the HIV-1 proteome reflects its competitiveness for presentation [33]. Predictions of HLA:peptide binding affinity were made for all 8- to 11mer peptides from the whole HIV-1 (NL4-3) genome using NetMHCpan v2.8. Peptides were ranked by affinity and, for each HLA allele, the highest ranking peptide containing the amino acid polymorphism is plotted. The horizontal line indicates rank 10. It can be seen that, with the exception of one HLA molecule which binds a peptide containing the polymorphism in Env, the variant peptides are very poor competitors for binding.
(PNG)

S2 Fig Enrichment of variant strain in KIR+ and KIR- individuals: Experimental observation and theoretical prediction. Predicted variant strain enrichment based on the more generous definition of the frequency of selecting HLAs (Table 1, column B) obtained if we relax our definition of what KIRs can bind. Grey dots: predicted enrichments obtained by randomly varying parameters within the range given in S3 Table, red circle: the observed enrichment. Even with this generous definition of a selecting HLA there are no parameter combinations which can match the experimental data for 4 of the 6 variants considered. Panels (**A-F**) correspond with panels B-G from Fig 2. i.e. (**A-E**) Fraction of variant-infected individuals in the *KIR2DL2+* and *KIR2DL2–* population for 100,000 randomly chosen parameter values. Grey dots: model predictions, red circle: observation reported by Alter et al. **A**: Env, **B**: Vpu, **C**: Gag, **D**: Nef, **E**: Tat. (**F**) Predicted and observed variant enrichment for the *KIR2DL3*-associated polymorphism in Vpu (3).
(TIF)

S3 Fig Impact of varying the fraction of selecting HLAs. Predicted fraction of variant-infected individuals in the *KIR2DL2*^**+**^ and *KIR2DL2*^**–**^ population for different fractions of selecting HLAs (*f*_*H*_) in the population. The fraction of selecting HLAs was varied (represented by different colours). The black circles represent the experimentally observed variant enrichment for the 6 variants. In all cases the observed enrichment can be predicted but only if the fraction of selectors is significantly higher than observed in the population.
(PNG)

S4 Fig Comparison of variant and wild type binding. Predicted binding of all 8-, 9-, 10-, 11-mers containing the variant position to all KIR ligating HLA molecules (i.e. all HLA C molecules, B*73:01 and B*46:01 for the *KIR2DL2*-associated variants and all HLA C1 molecules, B*73:01 and B*46:01 for the *KIR2DL3*-associated variant). The blue lines denote the point corresponding to 500nM (1-log_50,000_(500) = 0.426), peptide-HLA interactions below this threshold are considered to be non-binding. The red line is the line of equality (x = y).
(TIF)

## References

[1] Vilches C, Parham P. KIR: diverse, rapidly evolving receptors of innate and adaptive immunity. Annual review of immunology. 2002;20:217–51. 10.1146/annurev.immunol.20.092501.134942 11861603

[2] Malnati MS, Peruzzi M, Parker KC, Biddison WE, Ciccone E, Moretta A, et al Peptide specificity in the recognition of MHC class I by natural killer cell clones. Science (New York, NY. 1995;267(5200):1016–8. Epub 1995/02/17. . 7863326 10.1126/science.7863326

[3] Peruzzi M, Parker KC, Long EO, Malnati MS. Peptide sequence requirements for the recognition of HLA-B*2705 by specific natural killer cells. J Immunol. 1996;157(8):3350–6. . 8871631

[4] Rajagopalan S, Long EO. The direct binding of a p58 killer cell inhibitory receptor to human histocompatibility leukocyte antigen (HLA)-Cw4 exhibits peptide selectivity. The Journal of experimental medicine. 1997;185(8):1523–8. Epub 1997/04/21. ; 9126935 10.1084/jem.185.8.1523 PMC2196286

[5] Boyington JC, Motyka SA, Schuck P, Brooks AG, Sun PD. Crystal structure of an NK cell immunoglobulin-like receptor in complex with its class I MHC ligand. Nature. 2000;405(6786):537–43. Epub 2000/06/13. 10.1038/35014520 . 10850706

[6] Fadda L, Borhis G, Ahmed P, Cheent K, Pageon SV, Cazaly A, et al Peptide antagonism as a mechanism for NK cell activation. Proc Natl Acad Sci U S A. 2010;107(22):10160–5. 10.1073/pnas.0913745107 20439706 PMC2890497

[7] Fadda L, O'Connor GM, Kumar S, Piechocka-Troch A, Gardiner CM, Carrington M, et al Common HIV-1 Peptide Variants Mediate Differential Binding of KIR3DL1 to HLA-Bw4 Molecules. Journal of Virology. 2011;85(12):5970–4. 10.1128/JVI.00412-11 21471246 PMC3126328

[8] Van Teijlingen NH, HÖLzemer A, Rner CK, GarcÍA-BeltrÁN WF, Schafer J, Fadda L, et al Sequence variations in HIV-1 p24 Gag-derived epitopes can alter binding of KIR2DL2 to HLA- C*03:04 and modulate primary NK cell function. AIDS (London, England). 2014;28(10):1399–408. 10.1097/QAD.0000000000000284 24785948 PMC4453925

[9] Matusali G, Potesta M, Santoni A, Cerboni C, Doria M. The human immunodeficiency virus type 1 Nef and Vpu proteins downregulate the natural killer cell-activating ligand PVR. J Virol. 2012;86(8):4496–504. 10.1128/JVI.05788-11 ; 22301152 PMC3318642

[10] Cerboni C, Neri F, Casartelli N, Zingoni A, Cosman D, Rossi P, et al Human immunodeficiency virus 1 Nef protein downmodulates the ligands of the activating receptor NKG2D and inhibits natural killer cell-mediated cytotoxicity. J Gen Virol. 2007;88(Pt 1):242–50. 10.1099/vir.0.82125-0 . 17170457

[11] Cohen GB, Gandhi RT, Davis DM, Mandelboim O, Chen BK, Strominger JL, et al The selective downregulation of class I major histocompatibility complex proteins by HIV-1 protects HIV-infected cells from NK cells. Immunity. 1999;10(6):661–71. . 10403641 10.1016/s1074-7613(00)80065-5

[12] Matusali G, Tchidjou HK, Pontrelli G, Bernardi S, D'Ettorre G, Vullo V, et al Soluble ligands for the NKG2D receptor are released during HIV-1 infection and impair NKG2D expression and cytotoxicity of NK cells. FASEB J. 2013;27(6):2440–50. 10.1096/fj.12-223057 . 23395909

[13] Martin MP, Gao X, Lee J-H, Nelson GW, Detels R, Goedert JJ, et al Epistatic interaction between KIR3DS1 and HLA-B delays the progression to AIDS. Nature genetics. 2002;31(4):429–34. 10.1038/ng934 12134147

[14] Pelak K, Need AC, Fellay J, Shianna KV, Feng S, Urban TJ, et al Copy number variation of KIR genes influences HIV-1 control. PLoS biology. 2011;9(11):e1001208 10.1371/journal.pbio.1001208 22140359 PMC3226550

[15] Martin MP, Qi Y, Gao X, Yamada E, Martin JN, Pereyra F, et al Innate partnership of HLA-B and KIR3DL1 subtypes against HIV-1. Nature genetics. 2007;39(6):733–40. 10.1038/ng2035 17496894 PMC4135476

[16] Boulet S, Sharafi S, Simic N, Bruneau J, Routy JP, Tsoukas CM, et al Increased proportion of KIR3DS1 homozygotes in HIV-exposed uninfected individuals. AIDS. 2008;22(5):595–9. 10.1097/QAD.0b013e3282f56b23 . 18317000

[17] Boulet S, Kleyman M, Kim JY, Kamya P, Sharafi S, Simic N, et al A combined genotype of KIR3DL1 high expressing alleles and HLA-B*57 is associated with a reduced risk of HIV infection. AIDS. 2008;22(12):1487–91. 10.1097/QAD.0b013e3282ffde7e . 18614872

[18] Haynes BF, Gilbert PB, McElrath MJ, Zolla-Pazner S, Tomaras GD, Alam SM, et al Immune-Correlates Analysis of an HIV-1 Vaccine Efficacy Trial. New England Journal of Medicine. 2012;366(14):1275–86. 10.1056/NEJMoa1113425 . 22475592 PMC3371689

[19] Alter G, Heckerman D, Schneidewind A, Fadda L, Kadie CM, Carlson JM, et al HIV-1 adaptation to NK-cell-mediated immune pressure. Nature. 2011;476(7358):96–100. 10.1038/nature10237 21814282 PMC3194000

[20] Jost S, Altfeld M. Control of Human Viral Infections by Natural Killer Cells. Annual review of immunology. 2013;31(1):163–94. 10.1146/annurev-immunol-032712-100001 . 23298212

[21] Jost S, Altfeld M. Evasion from NK cell-mediated immune responses by HIV-1. Microbes Infect. 2012;14(11):904–15. 10.1016/j.micinf.2012.05.001 ; 22626930 PMC3432664

[22] Holzemer A, Thobakgale CF, Jimenez Cruz CA, Garcia-Beltran WF, Carlson JM, van Teijlingen NH, et al Selection of an HLA-C*03:04-Restricted HIV-1 p24 Gag Sequence Variant Is Associated with Viral Escape from KIR2DL3+ Natural Killer Cells: Data from an Observational Cohort in South Africa. PLoS Med. 2015;12(11):e1001900; discussion e. 10.1371/journal.pmed.1001900 ; 26575988 PMC4648589

[23] Goulder PJ, Phillips RE, Colbert RA, McAdam S, Ogg G, Nowak MA, et al Late escape from an immunodominant cytotoxic T-lymphocyte response associated with progression to AIDS. Nature medicine. 1997;3(2):212–7. . 9018241 10.1038/nm0297-212

[24] Phillips RE, Rowland-Jones S, Nixon DF, Gotch FM, Edwards JP, Ogunlesi AO, et al Human immunodeficiency virus genetic variation that can escape cytotoxic T cell recognition. Nature. 1991;354(6353):453–9. 10.1038/354453a0 1721107

[25] Asquith B, Edwards CT, Lipsitch M, McLean AR. Inefficient cytotoxic T lymphocyte-mediated killing of HIV-1-infected cells in vivo. PLoS biology. 2006;4(4):e90 10.1371/journal.pbio.0040090 ; 16515366 PMC1395353

[26] Moesta AK, Norman PJ, Yawata M, Yawata N, Gleimer M, Parham P. Synergistic polymorphism at two positions distal to the ligand-binding site makes KIR2DL2 a stronger receptor for HLA-C than KIR2DL3. J Immunol. 2008;180(6):3969–79. . 18322206 10.4049/jimmunol.180.6.3969

[27] Hansasuta P, Dong T, Thananchai H, Weekes M, Willberg C, Aldemir H, et al Recognition of HLA-A3 and HLA-A11 by KIR3DL2 is peptide-specific. European journal of immunology. 2004;34(6):1673–9. 10.1002/eji.200425089 . 15162437

[28] Thananchai H, Gillespie G, Martin MP, Bashirova A, Yawata N, Yawata M, et al Cutting Edge: Allele-specific and peptide-dependent interactions between KIR3DL1 and HLA-A and HLA-B. J Immunol. 2007;178(1):33–7. . 17182537 10.4049/jimmunol.178.1.33

[29] Thananchai H, Makadzange T, Maenaka K, Kuroki K, Peng Y, Conlon C, et al Reciprocal recognition of an HLA-Cw4-restricted HIV-1 gp120 epitope by CD8+ T cells and NK cells. AIDS. 2009;23(2):189–93. 10.1097/QAD.0b013e32831fb55a . 19098488

[30] Maiers M, Gragert L, Klitz W. High-resolution HLA alleles and haplotypes in the United States population (vol 68, pg 779, 2007). Human immunology. 2008;69(2):141-. 10.1016/j.humimm.2007.04.005 17869653

[31] US Centers for Disease control and Prevention. Diagnoses of HIV Infection in the United States and Dependent Areas, 2000. HIV Surveillance Report. 2000;12.

[32] Hoof I, Peters B, Sidney J, Pedersen L, Sette A, Lund O, et al NetMHCpan, a method for MHC class I binding prediction beyond humans. Immunogenetics. 2009;61(1):1–13. 10.1007/s00251-008-0341-z 19002680 PMC3319061

[33] Borghans JAM, Mølgaard A, de Boer RJ, Keşmir C. HLA Alleles Associated with Slow Progression to AIDS Truly Prefer to Present HIV-1 p24. PLoS ONE. 2007;2(9):e920–e. 10.1371/journal.pone.0000920 17878955 PMC1976389

[34] Peters B, Bui H-H, Frankild S, Nielson M, Lundegaard C, Kostem E, et al A community resource benchmarking predictions of peptide binding to MHC-I molecules. PLoS Comput Biol. 2006;2(6):e65 10.1371/journal.pcbi.0020065 16789818 PMC1475712

[35] Rasmussen M, Harndahl M, Stryhn A, Boucherma R, Nielsen LL, Lemonnier FA, et al Uncovering the peptide-binding specificities of HLA-C: a general strategy to determine the specificity of any MHC class I molecule. J Immunol. 2014;193(10):4790–802. 10.4049/jimmunol.1401689 ; 25311805 PMC4226424

[36] Harndahl M, Rasmussen M, Roder G, Buus S. Real-time, high-throughput measurements of peptide-MHC-I dissociation using a scintillation proximity assay. Journal of immunological methods. 2011;374(1–2):5–12. Epub 2010/11/04. 10.1016/j.jim.2010.10.012 ; 21044632 PMC4341823

[37] Harndahl M, Rasmussen M, Roder G, Dalgaard Pedersen I, Sorensen M, Nielsen M, et al Peptide-MHC class I stability is a better predictor than peptide affinity of CTL immunogenicity. European journal of immunology. 2012;42(6):1405–16. Epub 2012/06/09. 10.1002/eji.201141774 . 22678897

[38] Frater AJ, Brown H, Oxenius A, Gunthard HF, Hirschel B, Robinson N, et al Effective T Cell Responses Select HIV-1 Mutants and Slow Disease Progression. J Virol. 2007;81(12):6742–51. 10.1128/JVI.00022-07 17409157 PMC1900110

[39] Payne RP, Kloverpris H, Sacha JB, Brumme Z, Brumme C, Buus S, et al Efficacious early antiviral activity of HIV Gag- and Pol-specific HLA-B 2705-restricted CD8+ T cells. J Virol. 2010;84(20):10543–57. Epub 2010/08/06. 10.1128/JVI.00793-10 ; 20686036 PMC2950555

[40] Moore CB, John M, James IR, Christiansen FT, Witt CS, Mallal SA. Evidence of HIV-1 adaptation to HLA-restricted immune responses at a population level. Science (New York, NY. 2002;296(5572):1439–43. 10.1126/science.1069660 12029127

[41] Le Gall S, Stamegna P, Walker BD. Portable flanking sequences modulate CTL epitope processing. The Journal of clinical investigation. 2007;117(11):3563–75. Epub 2007/11/03. 10.1172/JCI32047 ; 17975674 PMC2045603

[42] Nielsen M, Lundegaard C, Blicher T, Lamberth K, Harndahl M, Justesen S, et al NetMHCpan, a Method for Quantitative Predictions of Peptide Binding to Any HLA-A and -B Locus Protein of Known Sequence. PLoS One. 2007;2(8):e796 10.1371/journal.pone.0000796 17726526 PMC1949492

[43] Zhang H, Lundegaard C, Nielsen M. Pan-specific MHC class I predictors: a benchmark of HLA class I pan-specific prediction methods. Bioinformatics. 2009;25(1):83–9. 10.1093/bioinformatics/btn579 18996943 PMC2638932

[44] Hirayasu K, Arase H. Functional and genetic diversity of leukocyte immunoglobulin-like receptor and implication for disease associations. Journal of human genetics. 2015;60(11):703–8. Epub 2015/06/05. 10.1038/jhg.2015.64 . 26040207

[45] Bashirova AA, Martin-Gayo E, Jones DC, Qi Y, Apps R, Gao X, et al LILRB2 interaction with HLA class I correlates with control of HIV-1 infection. PLoS Genet. 2014;10(3):e1004196 Epub 2014/03/08. 10.1371/journal.pgen.1004196 ; 24603468 PMC3945438

[46] Norman PJ, Cook MA, Carey BS, Carrington CV, Verity DH, Hameed K, et al SNP haplotypes and allele frequencies show evidence for disruptive and balancing selection in the human leukocyte receptor complex. Immunogenetics. 2004;56(4):225–37. Epub 2004/06/09. 10.1007/s00251-004-0674-1 . 15185041

[47] Liepe J, Marino F, Sidney J, Jeko A, Bunting DE, Sette A, et al A large fraction of HLA class I ligands are proteasome-generated spliced peptides. Science. 2016;354(6310):354–8. 10.1126/science.aaf4384 . 27846572

[48] Apps R, Del Prete Gregory Q, Chatterjee P, Lara A, Brumme Zabrina L, Brockman Mark A, et al HIV-1 Vpu Mediates HLA-C Downregulation. Cell Host & Microbe. 2016;19(5):686–95. 10.1016/j.chom.2016.04.005. 27173934 PMC4904791

[49] Heckerman D, Kadie C, Listgarten J. Leveraging information across HLA alleles/supertypes improves epitope prediction. J Comput Biol. 2007;14(6):736–46. 10.1089/cmb.2007.R013 17691891

[50] US Centers for Disease control and Prevention. Diagnoses of HIV Infection in the United States and Dependent Areas, 2013. HIV Surveillance Report. 2013;25.

[51] Fryer HR, Frater J, Duda A, Roberts MG, Phillips RE, McLean AR. Modelling the evolution and spread of HIV immune escape mutants. PLoS Pathog. 2010;6(11):e1001196 10.1371/journal.ppat.1001196 21124991 PMC2987822

[52] Hilton HG, Vago L, Older Aguilar AM, Moesta AK, Graef T, Abi-Rached L, et al Mutation at positively selected positions in the binding site for HLA-C shows that KIR2DL1 is a more refined but less adaptable NK cell receptor than KIR2DL3. J Immunol. 2012;189(3):1418–30. 10.4049/jimmunol.1100431 . 22772445 PMC3439511

