# Supplementary material for: HIV-1 adaptation to NK cell-mediated immune pressure
Source: PLoS Pathog. 2017 Jun 5;13(6):e1006361. doi: 10.1371/journal.ppat.1006361 (PMC5472325; doi:10.1371/journal.ppat.1006361)
Supplement: S1 Text — (DOCX) [file ppat.1006361.s001.docx]

**Supplementary Text S1. Selecting HLA molecules: an example**

As an example of the various definitions of a selecting HLA molecule employed in Table 1 consider the *KIR2DL2*-associated variant Gag 138L.

**Column A**

We say an HLA molecule is “selecting” if 1) the HLA molecule ligates the relevant iKIR and 2i) the variant peptide was predicted to bind the HLA molecule and had a polymorphism in a position that would interact with the KIR receptor (PC-1 or PC-2) or 2ii) the HLA molecule binds none of the wild type peptides and at least one of the variant peptides containing the variant position.

So an HLA molecule is considered to be selecting if it is any HLA-C molecule, or HLA B*4601 or HLA B*7301 which

Either binds any of the following peptides:

PIVQN**L**QG

YPIVQN**L**Q

YPIVQN**L**QG

NYPIVQN**L**Q

NYPIVQN**L**QG

QNYPIVQN**L**Q

QNYPIVQN**L**QG

SQNYPIVQN**L**Q

Or binds any of the following variant peptides but not the wild type

**L**QGQMVHQ

N**L**QGQMVH

QN**L**QGQMV

VQN**L**QGQM

IVQN**L**QGQ

PIVQN**L**QG

YPIVQN**L**Q

NYPIVQN**L**

**L**QGQMVHQA

N**L**QGQMVHQ

QN**L**QGQMVH

VQN**L**QGQMV

IVQN**L**QGQM

PIVQN**L**QGQ

YPIVQN**L**QG

NYPIVQN**L**Q

QNYPIVQN**L**

**L**QGQMVHQAI

N**L**QGQMVHQA

QN**L**QGQMVHQ

VQN**L**QGQMVH

IVQN**L**QGQMV

PIVQN**L**QGQM

YPIVQN**L**QGQ

NYPIVQN**L**QG

QNYPIVQN**L**Q

SQNYPIVQN**L**

**L**QGQMVHQAIS

N**L**QGQMVHQAI

QN**L**QGQMVHQA

VQN**L**QGQMVHQ

IVQN**L**QGQMVH

PIVQN**L**QGQMV

YPIVQN**L**QGQM

NYPIVQN**L**QGQ

QNYPIVQN**L**QG

SQNYPIVQN**L**Q

VSQNYPIVQN**L**

**Column B**

As above but any polymorphism in a non-anchor position is assumed to interact with the KIR receptor. So an HLA molecule is considered to be selecting if it is any HLA-C molecule , or HLA B*4601 or HLA B*7301 which

Either binds any of the following peptides:

**L**QGQMVHQ

QN**L**QGQMV

VQN**L**QGQM

IVQN**L**QGQ

PIVQN**L**QG

YPIVQN**L**Q

**L**QGQMVHQA

QN**L**QGQMVH

VQN**L**QGQMV

IVQN**L**QGQM

PIVQN**L**QGQ

YPIVQN**L**QG

NYPIVQN**L**Q

**L**QGQMVHQAI

QN**L**QGQMVHQ

VQN**L**QGQMVH

IVQN**L**QGQMV

PIVQN**L**QGQM

YPIVQN**L**QGQ

NYPIVQN**L**QG

QNYPIVQN**L**Q

**L**QGQMVHQAIS

QN**L**QGQMVHQA

VQN**L**QGQMVHQ

IVQN**L**QGQMVH

PIVQN**L**QGQMV

YPIVQN**L**QGQM

NYPIVQN**L**QGQ

QNYPIVQN**L**QG

SQNYPIVQN**L**Q

Or binds any of the following variant peptides but not the wild type

**L**QGQMVHQ

N**L**QGQMVH

QN**L**QGQMV

VQN**L**QGQM

IVQN**L**QGQ

PIVQN**L**QG

YPIVQN**L**Q

NYPIVQN**L**

**L**QGQMVHQA

N**L**QGQMVHQ

QN**L**QGQMVH

VQN**L**QGQMV

IVQN**L**QGQM

PIVQN**L**QGQ

YPIVQN**L**QG

NYPIVQN**L**Q

QNYPIVQN**L**

**L**QGQMVHQAI

N**L**QGQMVHQA

QN**L**QGQMVHQ

VQN**L**QGQMVH

IVQN**L**QGQMV

PIVQN**L**QGQM

YPIVQN**L**QGQ

NYPIVQN**L**QG

QNYPIVQN**L**Q

SQNYPIVQN**L**

**L**QGQMVHQAIS

N**L**QGQMVHQAI

QN**L**QGQMVHQA

VQN**L**QGQMVHQ

IVQN**L**QGQMVH

PIVQN**L**QGQMV

YPIVQN**L**QGQM

NYPIVQN**L**QGQ

QNYPIVQN**L**QG

SQNYPIVQN**L**Q

VSQNYPIVQN**L**

**Column C**

As for column A but restricted to 9mers only.

**Column D**

As for column A but restricted to the HLA-C1 alleles, HLA B*4601 and HLA B*7301 only.

Note that since we were unable to reliably predict whether an amino acid change enhances KIR signalling we make the generous assumption that for **any** of the peptides listed above the 138L mutation will enhance signalling. E.g under definition B, if HLA-C*01:02 binds **L**QGQMVHQ and HLA-C*03:04 binds NYPIVQN**L**QGQ both variant peptides are assumed to enhance KIR2DL2 signalling compared to wild type (even though the mutation is at different relative positions in the peptide and will likely contact KIR2DL2 differently). Both HLA-C*01:02 and HLA-C*03:04 would be assumed to be selecting HLA molecules. This generous assumption will lead to an overestimate of *f_H_*, i.e. we are erring on the side of caution.
