# Supplementary material for: HIV-1 adaptation to NK cell-mediated immune pressure
Source: PLoS Pathog. 2017 Jun 5;13(6):e1006361. doi: 10.1371/journal.ppat.1006361 (PMC5472325; doi:10.1371/journal.ppat.1006361)
Supplement: S2 Text — (DOCX) [file ppat.1006361.s002.docx]

**S2 Text: Additional analysis**

# Definition of wild type and variant virus.

Alter *et al* [1] divide their cohort into three groups: individuals infected with WT virus, variant virus and “other” virus. This last group are a considerable proportion of the cohort (11-34%). Whether individuals from this third group should be considered V- or WT-infected could affect the reported enrichment. We used the PMBEC similarity matrix [2], specifically developed to quantify amino acid similarity in peptide-MHC binding interactions, to classify substitutions other than those defined as WT or V:

**Protein position WT V other absolute covariance assigned to**

**WT V**

Vpu 71 R M L -0.07 0.08 **V**

Q 0.01 0 WT

N -0.03 0.01 V

V -0.09 -0.01 **V**

G -0.02 -0.03 WT

Vpu 74 L H D -0.03 -0.02 V

R -0.07 0.11 **V**

Env 17 G W R -0.02 -0.06 **WT**

M -0.03 0.02 V

20 L M T -0.03 -.05 **WT**

V 0.05 -0.01 **WT**

Gag 138 I L M 0.06 0.08 V

V 0.09 0.05 **WT**

A 0 -0.04 WT

Nef 9 K S E -0.07 -0.02 **V**

R 0.22 0.02 **WT**

N -0.03 0.02 V

C -0.05 -0.02 V

L -0.02 -0.06 **WT**

P -0.05 -0.01 V

I -0.04 -0.06 **WT**

G -0.01 0.03 V

W -0.06 -0.04 **V**

The assignments in **bold** are assignments with a high similarity or dissimilarity (<-0.05 or >0.05) for either WT or V.

Vpu and Env contain polymorphisms at two positions. If one of these positions contains the AA present in or most similar to WT and the other the AA present in or most similar to V, assignment to either strain is ambiguous. We considered two options: 1) the whole sequence is assigned to WT strain, 2) the whole sequence is assigned V strain. In this way we obtained a range for the proportion WT and V infected in the cohort for these two proteins.

We then used these assignments of WT and variant virus to recalculate enrichment in the *KIR2DL2*^+^ and *KIR2DL2*^–^ population:

|  | *KIR2DL2*^+^ | *KIR2DL2*^–^ | *KIR2DL2*^+^ | *KIR2DL2*^–^ |
| --- | --- | --- | --- | --- |
|  | Vpu |  | Env |  |
| Original | 0.74 | 0.32 | 0.85 | 0.43 |
| PMBEC strict | 0.74 | 0.34 | 0.85 | 0.43 |
| PMBEC generous | 0.87 | 0.48 | 0.85 | 0.45 |
|  | Gag |  | Nef |  |
| Original | 0.68 | 0.57 | 0.82 | 0.59 |
| PMBEC strict | 0.94 | 0.75 | 0.82 | 0.59 |
| PMBEC generous | 0.94 | 0.75 | 0.91 | 0.69 |

We found that these enrichments are still far from the predicted enrichments and conclude that changing the assignment of the third group of variants does not help to explain the discrepancy between the observed and expected variant enrichment.

1. Alter G, Heckerman D, Schneidewind A, Fadda L, Kadie CM, Carlson JM, et al. HIV-1 adaptation to NK-cell-mediated immune pressure. Nature. 2011;476(7358):96-100. PubMed PMID: 21814282.

2. Kim Y, Sidney J, Pinilla C, Sette A, Peters B. Derivation of an amino acid similarity matrix for peptide: MHC binding and its application as a Bayesian prior. BMC bioinformatics. 2009;10:394. Epub 2009/12/02. doi: 10.1186/1471-2105-10-394. PubMed PMID: 19948066; PubMed Central PMCID: PMCPMC2790471.
