# Supplementary material for: HIV-1 adaptation to NK cell-mediated immune pressure
Source: PLoS Pathog. 2017 Jun 5;13(6):e1006361. doi: 10.1371/journal.ppat.1006361 (PMC5472325; doi:10.1371/journal.ppat.1006361)
Supplement: S1 Table — An alternative definition of whether a peptide binds an HLA molecule is the rank of its binding affinity compared to the binding affinity of all peptides from the viral proteome. The idea is that only viral peptides in the top 5 or 10 binders for a single HLA molecule will be competitive [33]. Table below shows the median and minimum rank of the variant peptide with the highest affinity relative to the whole HIV-1 genome, the number of HLA alleles for which the highest ranking peptide falls into the top 5 or top 10 of the whole HIV-genome and the carrier frequency of selecting HLAs (fH) based on this definition. (DOCX) [file ppat.1006361.s003.docx]

## **S1 Table. Fraction of selecting HLAs based on rank definition of an epitope (NetMHCpan).**

An alternative definition of whether a peptide binds an HLA molecule is the rank of its binding affinity compared to the binding affinity of all peptides from the viral proteome. The idea is that only viral peptides in the top 5 or 10 binders for a single HLA molecule will be competitive [[30](#_ENREF_30)]. Table below shows the median and minimum rank of the variant peptide with the highest affinity relative to the whole HIV-1 genome, the number of HLA alleles for which the highest ranking peptide falls into the top 5 or top 10 of the whole HIV-genome and the carrier frequency of selecting HLAs (*f_H_*) based on this definition.

| **Variant** | **Median rank** | **Minimum rank** | **Peptides in top 5** | **Peptides in top 10** | ***f_H_*** |
| --- | --- | --- | --- | --- | --- |
| Env(17/20) | 172 | 4 | 1 | 1 | 0.0004 |
| Vpu(71/74) | 234 | 52 | 0 | 0 | 0 |
| Gag(138) | 275 | 30 | 0 | 0 | 0 |
| Nef(9) | 158 | 21 | 0 | 0 | 0 |
| Tat(3) | 1387 | 170 | 0 | 0 | 0 |
| Vpu(3) | 258 | 64 | 0 | 0 | 0 |
