# Supplementary material for: HIV-1 adaptation to NK cell-mediated immune pressure
Source: PLoS Pathog. 2017 Jun 5;13(6):e1006361. doi: 10.1371/journal.ppat.1006361 (PMC5472325; doi:10.1371/journal.ppat.1006361)
Supplement: S2 Table — The carrier frequency of selecting HLAs (fH) was calculated using an alternative, independent epitope prediction algorithm Epipred. Results are tabulated below. (DOCX) [file ppat.1006361.s004.docx]

## **S2 Table. Fraction of selecting HLAs based on rank definition of an epitope (Epipred).**

The carrier frequency of selecting HLAs (*f_H_*) was calculated using an alternative, independent epitope prediction algorithm Epipred. Results are tabulated below.

| **Variant** | **Median rank** | **Minimum rank** | **Peptides in top5** | | **Peptides in top10** | ***f_H_*** |
| --- | --- | --- | --- | --- | --- | --- |
| Env(17/20) | 215 | 72 | 0 | 0 | | 0 |
| Vpu(71/74) | 57 | 19 | 0 | 0 | | 0 |
| Gag(138) | 228 | 136 | 0 | 0 | | 0 |
| Nef(9) | 84 | 35 | 0 | 0 | | 0 |
| Tat(3) | 71 | 13 | 0 | 0 | | 0 |
| Vpu(3) | 932 | 325 | 0 | 0 | | 0 |
