# Supplementary material for: HIV-1 adaptation to NK cell-mediated immune pressure
Source: PLoS Pathog. 2017 Jun 5;13(6):e1006361. doi: 10.1371/journal.ppat.1006361 (PMC5472325; doi:10.1371/journal.ppat.1006361)
Supplement: S3 Table — (DOCX) [file ppat.1006361.s005.docx]

## S3 Table. Parameter values for the model of viral evolution.

| **Symbol** | **Description** | **Range** | **Note** |
| --- | --- | --- | --- |
| t0 | time of first infection in US | 1940-1975 | 1 |
| T_cART_ | time of introduction of cART | 1996 | 1 |
| N(0) | size of US population at t0 | 1.3E8-2.3E8 | 2 |
|  | size of Swiss population at t0 | 4.6E6-6.3E6 | 3 |
|  | size of Canadian population at t0 | 1.2E7-2.3E7 | 4 |
|  | size of Australian population at t0 | 7.4E6-1.4E7 | 5 |
| k | fraction of *KIR2DL2*^+^ people in US | 0.4-0.6 | 6 |
|  | fraction of *KIR2DL3*^+^ people in US | 0.85-0.95 | 6 |
| μ | death rate HIV-1 seronegative people | 0.013-0.016 year^-1^ | 7 |
| B | birth rate in US | 4.2E6-7.1E6 year^-1^ | 8 |
|  | birth rate in Switzerland | 3.7E4-5.5E4 year^-1^ | 9 |
|  | birth rate in Canada | 2.5E5-4.2E5 year^-1^ | 4 |
|  | birth rate in Australia | 1.6E5-3.1E5 year^-1^ | 5 |
| α_,_α_1_,α_2_ | death rate HIV-seropositive people | different schemes | 10 |
| β_pre_ | transmission probability before introduction of cART | 0.45-0.55 year^-1^ | 11 |
| β_post_ | transmission probability after introduction of cART | 0.1-1 x β_pre_ year^-1^ | 11 |
| ϕ | Escape rate of variant strain | 0-12 year^-1^ | 12 |
| ψ | Reversion rate of variant strain | 0-1 year^-1^ | 12 |

^1^ Epidemiology of HIV/AIDS in the United States. Osmond 2003

^2,8^based on US census data [1]

^3,9^ from <http://www.bfs.admin.ch/bfs/portal/en/index/themen/01/02/blank/key/>

bevoelkerungsstand.html

^4^ from http://www.statcan.gc.ca/pub/98-187-x/4151287-eng.htm

^5^ from <http://www.abs.gov.au/AUSSTATS/abs@.nsf/DetailsPage/>3105.0.65.0012014?

OpenDocument

^6^ allellefrequencies.net

^7^ based on life expectancy of 60-80 yrs [2]

^10^ Two different mortality schemes were considered both based on average mortality post-combination antiretroviral therapy (cART) of 0.025-0.1 per year, increased by a factor 1-5 pre-cART:

Scheme 1. A selector with escape variant virus has a mortality disadvantage compared to all other individuals (α_1_ =α <α_2_).

|  | Pre-cART | Post-cART |
| --- | --- | --- |
| α_1_ | (1-5)x0.025-0.1 yr^-1^ | 0.025-0.1 yr^-1^ |
| α_2_ | (1-5)x(1-5)x0.025-0.1 yr^-1^ | (1-5)x0.025-0.1 yr^-1^ |
| α | (1-5)x0.025-0.1 yr^-1^ | 0.025-0.1 yr^-1^ |

Base post-cART mortality of 0.025-0.1 yr^-1^, increased by a factor 1-5 pre-cART and increased by a factor 1-5 if escape variant is present in a selector.

Scheme 2. A selector with wt virus has a mortality advantage compared to all other categories of HIV-1-infected individuals (α_1_<α_2_=α).

|  | Pre-cART | Post-cART |
| --- | --- | --- |
| α_1_ | 0.025-0.1 yr^-1^ | 0.025-0.1 yr^-1^ |
| α_2_ | (1-5)x0.025-0.1 yr^-1^ | 0.025-0.1 yr^-1^ |
| α | (1-5)x0.025-0.1 yr^-1^ | 0.025-0.1 yr^-1^ |

Post-cART mortality of 0.025-0.1 yr^-1^, increased by a factor 1-5 pre-cART and decreased by a factor 1-5 if wt is present in a selector.

^11^ set to obtain realistic size of infected population

^12^ escape rate and reversion rate were varied widely as these seemed to be the parameters most likely to yield the observed enrichment

1. Population Division UCB. Historical National Population Estimates: July 1, 1900 to July 1, 1999. <http://wwwcensusgov/popest/data/national/totals/pre-1980/tables/popclockesttxt>. 2000.

2. Service CR. CRS Report for Congress: Life Expectancy in the United States: August 16, 2006: BiblioGov; 2013.
