# Supplementary material for: HIV-1 adaptation to NK cell-mediated immune pressure
Source: PLoS Pathog. 2017 Jun 5;13(6):e1006361. doi: 10.1371/journal.ppat.1006361 (PMC5472325; doi:10.1371/journal.ppat.1006361)
Supplement: S4 Table — HLA ligands of many of the activating KIRS are unknown and/or contentious. We therefore considered all commonly assumed ligands (column “assumed HLA ligand”). KIR2DS5 is believed to have evolved from an activating C2 receptor but appears to have lost the capacity to bind HLA class I [52]; the two polymorphisms identified by Alter et al as associated with KIR2DS5 were therefore excluded from our analysis. Additionally, a third polymorphism identified by Alter et al (Env 46 KIR3DS1) was excluded as we were unable to identify the variant amino acid. (DOCX) [file ppat.1006361.s006.docx]

## S4 Table. Frequency of selecting HLAs for polymorphisms associated with activating KIR.

HLA ligands of many of the activating KIRs are unknown and/or contentious. We therefore considered all commonly assumed ligands (column “assumed HLA ligand”). KIR2DS5 is believed to have evolved from an activating C2 receptor but appears to have lost the capacity to bind HLA class I [1]; the two polymorphisms identified by Alter *et al* as associated with *KIR2DS5* were therefore excluded from our analysis. Additionally, a third polymorphism identified by Alter et al (Env 46 *KIR3DS1*) was excluded as we were unable to identify the variant amino acid.

| **Polymorphism position** | **KIR** | **assumed HLA ligand** | **Frequency of selecting HLAs (*f_H_*) A B** | |
| --- | --- | --- | --- | --- |
| Env 347 | 2DS1 | C | 0.0001 | 0.02 |
|  |  | C2 | 0.0001 | 0.0001 |
| Env 595 | 2DS1 | C | 0.03 | 0.03 |
|  |  | C2 | 0 | 0 |
| Gag 138 | 2DS2 | C | 0 | 0.02 |
|  |  | C1 | 0 | 0.02 |
| Gag389 | 3DS1 | Bw4 | 0.04 | 0.04 |
|  |  | Bw4-80I | 0 | 0.003 |
| Gag 479 | 2DS1 | C | 5.00E-05 | 5.00E-05 |
|  |  | C2 | 5.00E-05 | 5.00E-05 |
| Gag 93 | 2DS3 | C | 0.17 | 0.17 |
|  |  | C1 | 0.07 | 0.07 |
| Tat 28 | 2DS1 | C | 0.05 | 0.05 |
|  |  | C2 | 0.05 | 0.05 |
| Tat 3 | 3DS1 | Bw4 | 0.12 | 0.31 |
|  |  | Bw4-80I | 0.12 | 0.12 |
| Tat 3 | 2DS2 | C | 0 | 0 |
|  |  | C1 | 0 | 0 |
| Tat 9 | 2DS3 | C | 0 | 0 |
|  |  | C1 | 0 | 0 |
| Vpr 37 | 2DS3 | C | 0.77 | 0.77 |
|  |  | C1 | 0.47 | 0.47 |

1. Hilton HG, Vago L, Older Aguilar AM, Moesta AK, Graef T, Abi-Rached L, et al. Mutation at positively selected positions in the binding site for HLA-C shows that KIR2DL1 is a more refined but less adaptable NK cell receptor than KIR2DL3. J Immunol. 2012;189(3):1418-30.
