# Supplementary figures and images for: HIV-1 adaptation to NK cell-mediated immune pressure
Source: PLoS Pathog. 2017 Jun 5;13(6):e1006361. doi: 10.1371/journal.ppat.1006361 (PMC5472325; doi:10.1371/journal.ppat.1006361)

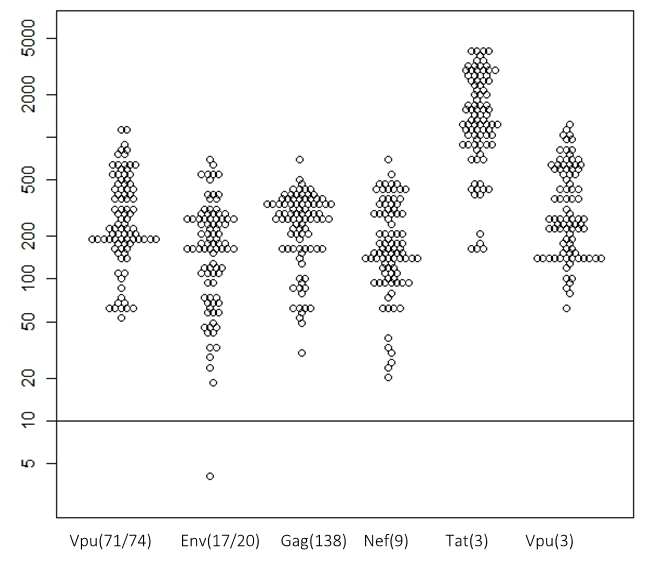

Supplement: S1 Fig — How strongly a peptide binds an HLA molecule relative to other peptides of the HIV-1 proteome reflects its competitiveness for presentation [33]. Predictions of HLA:peptide binding affinity were made for all 8- to 11mer peptides from the whole HIV-1 (NL4-3) genome using NetMHCpan v2.8. Peptides were ranked by affinity and, for each HLA allele, the highest ranking peptide containing the amino acid polymorphism is plotted. The horizontal line indicates rank 10. It can be seen that, with the exception of one HLA molecule which binds a peptide containing the polymorphism in Env, the variant peptides are very poor competitors for binding. (PNG) [file ppat.1006361.s007.png]

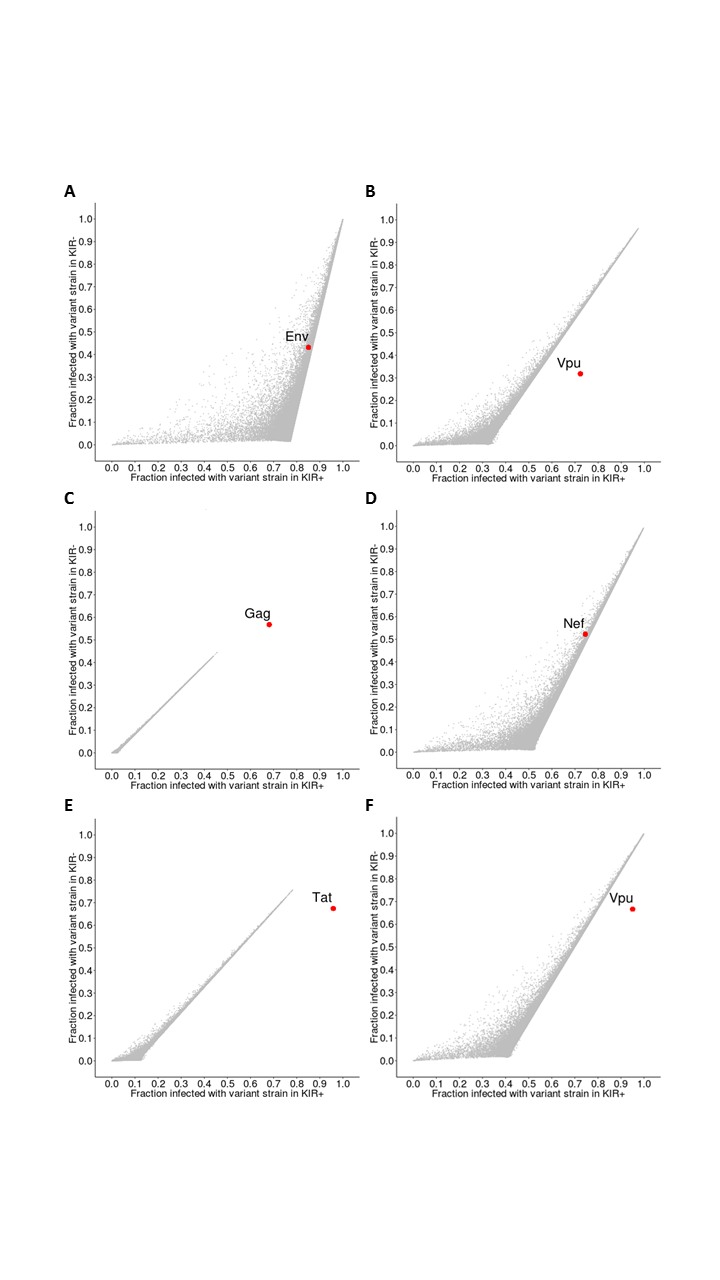

Supplement: S2 Fig — Predicted variant strain enrichment based on the more generous definition of the frequency of selecting HLAs (Table 1, column B) obtained if we relax our definition of what KIRs can bind. Grey dots: predicted enrichments obtained by randomly varying parameters within the range given in S3 Table, red circle: the observed enrichment. Even with this generous definition of a selecting HLA there are no parameter combinations which can match the experimental data for 4 of the 6 variants considered. Panels (A-F) correspond with panels B-G from Fig 2. i.e. (A-E) Fraction of variant-infected individuals in the KIR2DL2+ and KIR2DL2– population for 100,000 randomly chosen parameter values. Grey dots: model predictions, red circle: observation reported by Alter et al. A: Env, B: Vpu, C: Gag, D: Nef, E: Tat. (F) Predicted and observed variant enrichment for the KIR2DL3-associated polymorphism in Vpu (3). (TIF) [file ppat.1006361.s008.tif]

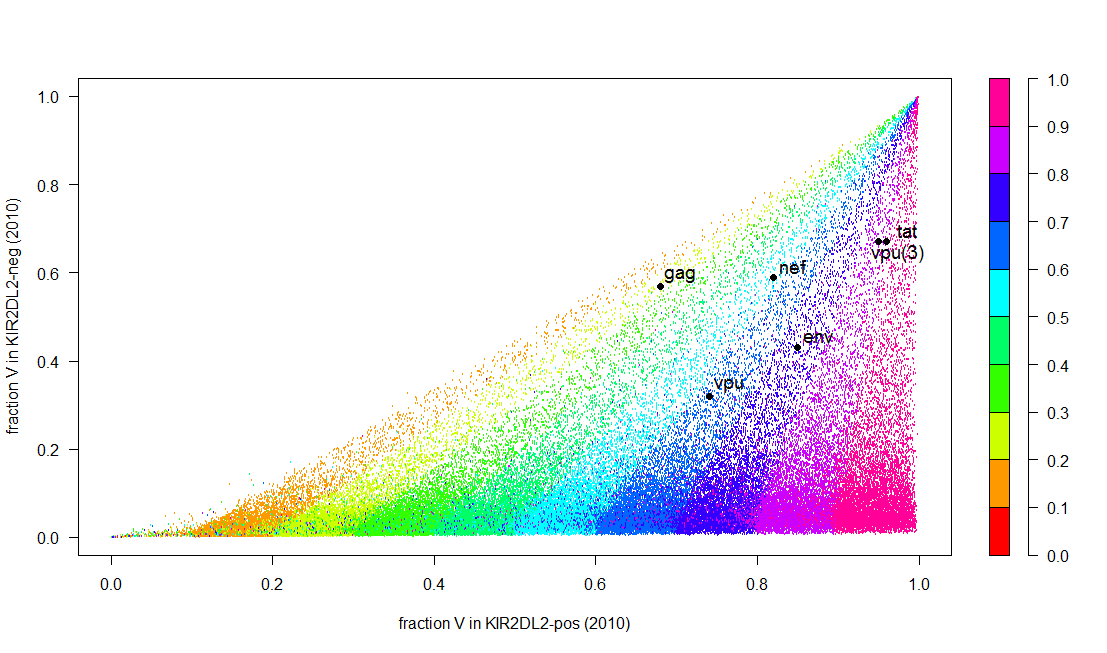

Supplement: S3 Fig — Predicted fraction of variant-infected individuals in the KIR2DL2+ and KIR2DL2– population for different fractions of selecting HLAs (fH) in the population. The fraction of selecting HLAs was varied (represented by different colours). The black circles represent the experimentally observed variant enrichment for the 6 variants. In all cases the observed enrichment can be predicted but only if the fraction of selectors is significantly higher than observed in the population. (PNG) [file ppat.1006361.s009.png]

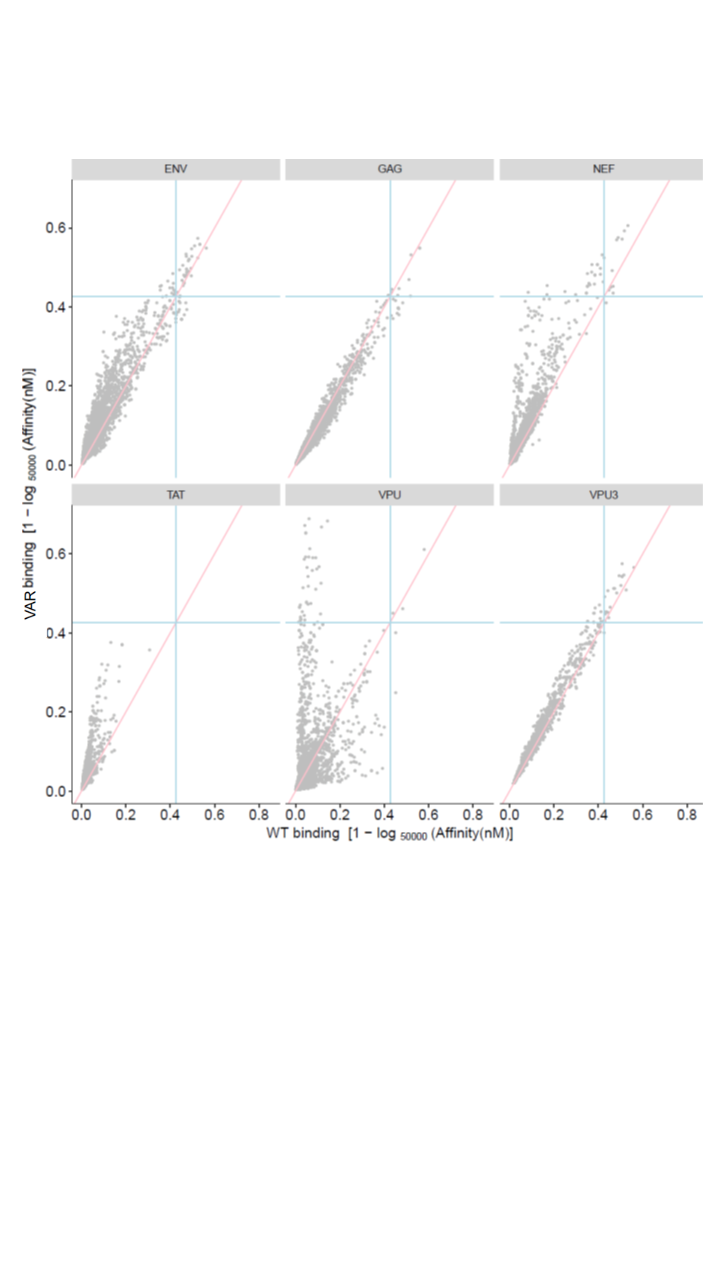

Supplement: S4 Fig — Predicted binding of all 8-, 9-, 10-, 11-mers containing the variant position to all KIR ligating HLA molecules (i.e. all HLA C molecules, B*73:01 and B*46:01 for the KIR2DL2-associated variants and all HLA C1 molecules, B*73:01 and B*46:01 for the KIR2DL3-associated variant). The blue lines denote the point corresponding to 500nM (1-log50,000(500) = 0.426), peptide-HLA interactions below this threshold are considered to be non-binding. The red line is the line of equality (x = y). (TIF) [file ppat.1006361.s010.tif]
